# Identification of Loci and Candidate Genes Responsible for Fiber Length in Upland Cotton (*Gossypium hirsutum* L.) via Association Mapping and Linkage Analyses

**DOI:** 10.3389/fpls.2019.00053

**Published:** 2019-02-05

**Authors:** Chi Zhang, Libei Li, Qibao Liu, Lijiao Gu, Jianqin Huang, Hengling Wei, Hantao Wang, Shuxun Yu

**Affiliations:** ^1^College of Agronomy, Northwest A&F University, Yangling, China; ^2^State Key Laboratory of Subtropical Silviculture, Zhejiang A & F University, Lin’an, China; ^3^State Key Laboratory of Cotton Biology, Institute of Cotton Research, Chinese Academy of Agricultural Sciences, Anyang, China

**Keywords:** upland cotton, fiber length, GWAS, QTL, sucrose synthesis

## Abstract

Fiber length (FL) is an important fiber quality trait in cotton. Although many fiber quality quantitative trait loci (QTL) responsible for FL have been identified, most cannot be applied to breeding programs, mainly due to unstable environments or large confidence intervals. In this study, we combined a genome-wide association study (GWAS) and linkage mapping to identify and validate high-quality QTLs responsible for FL. For the GWAS, we developed 93,250 high-quality single-nucleotide polymorphism (SNP) markers based on 355 accessions, and the FL was measured in eight different environments. For the linkage mapping, we constructed an *F*_2_ population from two extreme accessions. The high-density linkage maps spanned 3,848.29 cM, with an average marker interval of 1.41 cM. In total, 14 and 13 QTLs were identified in the association and linkage mapping analyses, respectively. Most importantly, a major QTL on chromosome D03 identified in both populations explained more than 10% of the phenotypic variation (PV). Furthermore, we found that a sucrose synthesis-related gene (*Gh_D03G1338*) was associated with FL in this QTL region. The RNA-seq data showed that *Gh_D03G1338* was highly expressed during the fiber development stage, and the qRT-PCR analysis showed significant expression differences between the long fiber and short fiber varieties. These results suggest that *Gh_D03G1338* may determine cotton fiber elongation by regulating the synthesis of sucrose. Favorable QTLs and candidate genes should be useful for increasing fiber quality in cotton breeding.

## Introduction

Cotton (*Gossypium* L.) is one of the most important cash crops and is extensively cultivated in more than 80 countries, having an annual global economic impact of approximately $500 billion and accounting for 2.5% of arable land worldwide (Chen et al., 2007). Upland cotton (*Gossypium hirsutum.* L) is the most important species due to its high yield and wide adaptability and is used as a raw material in the textile industry. The fiber quality is considered a key indicator for breeding programs, and tremendous breeding efforts have focused on fiber length (FL) to increase fiber quality (Said et al., 2015). FL is one of the most important and highly heritable fiber quality traits in cotton (Jamshed et al., 2016) and is directly related to its spinning performance, as longer fibers are typically better for manufacturing fine yarns. Over the last few decades, FL has been successfully used for genetic analysis, such as QTL mapping and association analysis, and more than 490 QTLs for FL have been reported (Said et al., 2015). For example, Liu et al. (2018) constructed a high-density genetic map containing 4,729 SNPs and 122 simple sequence repeat (SSR) markers with an average interval of 0.51 cM and anchored 36 QTLs for FL on 21 chromosomes in 9 environments. Ali et al. (2018) identified 20 QTLs related to FL in a RIL population derived from two cultivars (Yumian 1 and CA3084) with distinct genetic backgrounds, and 12 QTLs were detected in more than two environments. In addition, Huang et al. (2017) employed association mapping techniques, which are different from biparental linkage mapping, using 1,1975 high-quality SNP markers in a set of 503 upland cotton accessions and identified 11 highly favorable SNP alleles for FL. Thus, a better understanding of the genetic architecture of FL could help breeders develop varieties with longer fibers.

Molecular markers are powerful tools in QTL analyses of major traits and the identification of genomic loci that could be used in marker-assisted selection (MAS) breeding (Park et al., 2005). In the past few decades, molecular markers, including amplified fragment length polymorphisms (AFLPs) (Lacape et al., 2003), restriction fragment length polymorphisms (RFLPs) (Paterson et al., 1993), random amplified polymorphic DNAs (RAPDs) (Iqbal et al., 1997), sequence-related amplified polymorphisms (SRAPs) (Lin et al., 2003) and SSR markers (Blenda et al., 2006), have been widely used in cotton QTL mapping. However, compared with traditional molecular markers, SNPs are more efficient in revealing genetic changes in complex traits in association analyses and biparental QTL mapping because SNPs are widely distributed, highly polymorphic and can be obtained at a low cost in crop genomes (Van Tassell et al., 2008; Ganal et al., 2009). To date, genome-wide SNP discovery has been applied in multiple crops, including rice, maize, soybean, and oilseed rape. However, few QTLs have been discovered in cotton genetic studies using SNP markers compared with the number discovered in studies using traditional molecular markers (Said et al., 2015). For example, our laboratory previously published a high-density genetic map spanning 4,071.98 cM and identified 247 early-maturity QTLs based on restriction site-associated DNA sequencing (RAD-seq) (Jia et al., 2016). Subsequently, we used the genotyping by sequencing (GBS-seq) method to confirm a major QTL region on chromosome D03, providing valuable information for MAS breeding in early-maturity cotton (Li L. et al., 2017). Recently, a candidate gene responsible for plant height has been detected through association mapping in upland cotton accessions by using specific locus amplified fragment sequencing (SLAF-seq) (Su et al., 2018). Furthermore, the CottonSNP63K (Hinze et al., 2017) and CottonSNP80K (Cai et al., 2017) arrays for hybridization have become popular among QTL mapping and genome-wide association study (GWAS) analysis for the detection of QTLs responsible for fiber quality (Huang et al., 2017; Tan et al., 2018).

Genome-wide association study analyses have recently become a popular approach for revealing the genetic basis of quantitative phenotypic variation and identifying linkage markers for MAS breeding (Li et al., 2013; Mao et al., 2015; Yano et al., 2016). Compared with biparental linkage mapping, GWAS have the advantage of a higher resolution, allow for the identification of genes responsible for multiple traits and do not require the generation of a mapping population over a long period (Huang and Han, 2014). However, the substructure of a population can yield false-positive QTLs between markers and traits in a GWAS (Zhao et al., 2007). To overcome this deficiency, a new approach employing GWAS and QTL mapping to complement one another in the identification of major QTLs responsible for important agronomic traits has been used in various crops (Sonah et al., 2015; Zhao et al., 2015; Li X. et al., 2016; Sun et al., 2016). This method increases the confidence in the significant loci identified by GWAS and has been validated by QTL mapping. In addition, traditional linkage analysis is a powerful tool for analyzing pairs of alleles at a low resolution, and association mapping provides a high-resolution evaluation of numerous alleles with uneven statistical power (Wilson et al., 2004). Due to these advantages, this approach is more accurate and efficient for evaluating major QTLs harboring target genes responsible for important agronomic traits.

To obtain better insight into the underlying genetic mechanisms of FL in cotton, SLAF-seq and GBS-seq were used to discover genome-wide SNP markers in natural and biparental populations, respectively. The FL phenotype was evaluated in multiple environments. Then, we used both GWAS and linkage mapping to complement one another and identify reliable QTLs associated with variations in FL. The stable QTLs verified in this study may be useful for MAS or genomic selection and gene cloning in cotton breeding programs.

## Materials and Methods

### Mapping Population and Field Experiments

In total, 355 of the upland cotton cultivars, including 328 representative cultivars developed in China and 27 cultivars introduced from abroad, investigated in this study in the association analysis were obtained from cotton germplasm collections at our laboratory and the low-temperature germplasm gene bank of the Cotton Research Institute of the Chinese Academy of Agricultural Sciences (CRI-CAAS). Detailed information regarding all accessions is provided in Supplementary Table S1. The multiple environment evaluations were conducted between 2014 and 2016 in four different locations throughout China, including Anyang (36° 08′N, 114° 48′E), Shihezi (44° 31′N, 86° 01′E), Huanggang (30° 57′N, 114° 92′E), and Sanya (18° 36′N, 109° 17′E). Su et al. (2016) investigated four environments between 2014 and 2015 in Anyang and Shihezi. To enhance the accuracy of our phenotypic data, we added four environments and two additional locations to the total phenotypic data set (Table 1). Among the 355 accessions, we selected two commercial Chinese cotton cultivars bred by CRI-CAAS that display a significant difference in FL, i.e., the short fiber parent CRI50 is 27.52 mm, while the long fiber parent CRI60 is 31.22 mm. A segregation population was derived from a cross between CRI50 and CRI60 in the summer of 2014 to obtain F_1_ seeds in CRI-CAAS, Anyang, Henan Province, China (36° 08′N, 114° 48′E). In the winter of 2014, the F_1_ individual plants were self-pollinated, and seeds from 198 *F*_2_ individuals were harvested in Sanya, Hainan Province, China (18° 36′N, 109° 17′E). In 2015, *F*_2_ individual plants were self-pollinated, and *F*_2:3_ family seeds were harvested. All field experiments were performed in a randomized complete block design with three replications. The field management was performed according to standard local agronomic practices and cultivation conditions. Pesticides were used to control pests and diseases.

**Table 1:** Phenotypic variation in fiber length (FL) in the genome-wide association study and *F*_2_ (*F*_2:3_) populations.

Population	Environment	Minimum	Maximum	Mean	*SD*	Skewness	Kurtosis
GWAS	AY-2014	24.55	34.59	29.46	1.37	0.27	0.87
	AY-2015	23.25	34.40	29.05	1.72	-0.16	-0.19
	AY-2016	23.37	33.13	28.69	1.56	-0.01	0.09
	SHZ-2014	25.16	32.09	28.15	1.11	0.4	0.81
	SHZ-2015	24.07	32.27	27.77	1.53	0.02	-0.41
	SHZ-2016	24.45	35.50	29.45	1.59	0.41	0.93
	HG-2016	23.33	33.53	28.15	1.65	0.36	0.42
	SY-2016	22.60	34.05	28.34	1.74	0.09	0.46
*F*_2_	AY-2015	25.90	33.50	29.86	1.34	-0.13	0.41
*F*_2:3_	AY-2016	27.73	32.60	30.11	0.85	-0.17	0.36


### Phenotyping and Statistical Analysis

In total, 20 naturally opened bolls were hand-harvested from each line. The FL of approximately 10–15-g fiber samples was measured using an HVI-MF 100 instrument (User Technologies, Inc., USTER, Switzerland) at the Cotton Fiber Quality Supervision, Inspection and Testing Center of the Ministry of Agriculture, Anyang, China. The phenotypic data were analyzed using R software (R Core Team, 2013). The *P*-values of the correlation coefficients of the FL between each two environments were calculated with Pearson’s correlation test using the cor.test() function in (R Core Team, 2013). The broad-sense heritability was calculated using the methods described by Knapp (Knapp et al., 1985). In addition, the best linear unbiased prediction of fiber length (FL-BLUP) in each line in the eight environments was calculated for the GWAS analyses with the R package “lme4” (Bates et al., 2014).

### DNA Extraction and SNP Genotyping

For each cotton accession, the genomic DNA extraction was performed using the CTAB method proposed by Paterson et al. (1993) with modifications. The biparental populations were genotyped using a GBS-seq approach. GBS sequencing libraries were constructed for each accession based on a double digestion with the restriction enzymes *Mse* I and *NIa* III (New England Biolabs, NEB). The detailed protocols used for the library preparation and sequencing using the GBS strategy have been described by Zhou et al. (2016) and Li L. et al. (2017). The paired end 150-bp sequence reads in each library were generated using an Illumina HiSeq4000 (Illumina, San Diego, CA, United States). The clean reads from the parents and *F*_2_ individuals were aligned to the reference genome (TM-1) (Zhang et al., 2015) with Burrows–Wheeler Aligner (Li and Durbin, 2009). Only sequences with a mapping score of at least 20 and those aligning to the reference genome with fewer than two mismatches were used for the SNP discovery. The retained sequences were inputted into the Genome Analysis Toolkit software (McKenna et al., 2010), and a variant calling analysis was performed. The high-quality SNPs were filtered using vcftools (Danecek et al., 2011) and a Python script. The structural and functional annotations of the SNPs were analyzed using ANNOVAR (Wang et al., 2010) based on the GFF3 files of the *G. hirsutum* genome. Before the genetic map construction, all SNP markers were filtered using the criteria detailed by Li L. et al. (2017) to exclude those with more than 40% missing data in the progeny.

For the natural population, the SNP genotyping of the 355 accessions was performed using the SLAF-seq method (Sun et al., 2013). The genomic DNAs from each accession were incubated with *Rsa* I and *Hae* III (New England Biolabs, NEB). The sequencing of the population using the Illumina HiSeq2500 platform (Illumina, San Diego, CA, United States) generated 96.10 Gb of data with ∼80-bp, paired-end, clean reads. The SNP calling was performed using Genome Analysis Toolkit software (McKenna et al., 2010) and SAMtools (Li L. et al., 2017). Finally, the SNPs used for the GWAS were filtered using vcftools (Danecek et al., 2011) with a minor allele frequency (MAF) ≥ 0.05 and missing data < 0.2.

### Genome-Wide Associations and Candidate Gene Identification

For the GWAS analysis, TASSEL (Bradbury et al., 2007) was used to determine the association between the high-quality SNPs and FL-BLUP values calculated from the eight environments. A *P*-value < 2.5 × 10^-5^ was used as the threshold to determine whether a significant association with the markers existed. Candidate genes were identified in a significant LD block region on chromosome D03 (41.52–41.91 Mb). A Manhattan plot and a quantile-quantile plot were generated using the R package^1^ “CMplot”.

### Genetic Map Construction and QTL Analyses

A genetic map of the *F*_2_ populations was constructed using JoinMap 4.0 (Van Ooijen, 2006) with a regression approach and a logarithm of odds (LOD) threshold of 3–10. The recombinant ratio was converted to the genetic distance with the Kosambi map function (Kosambi, 2016). We divided the linkage groups according to their position on the *G. hirsutum* genome (Zhang et al., 2015), and the markers in each linkage group were then sorted with a Python script. The detection of the FL QTLs was performed using the software IciMapping with the composite interval mapping (CIM) model (Meng et al., 2015). An LOD threshold of 2.5 was used to identify the presence of significant QTLs. A graphic visualization of the linkage groups was created using the R package ggplot2 (Wickham, 2016).

### Gene Expression Level Analysis

The total RNA was extracted using an RNAprep Pure Plant Kit (Tiangen, China), and cDNA was reverse-transcribed with a PrimeScript^TM^ RT Reagent Kit and gDNA Eraser (TaKaRa, Japan). A real-time PCR detection system (7500 Real-Time PCR System, Applied Biosystems, Foster City, CA, United States) was used with the UltraSYBR Mixture (CWBIO, China). The actin transcript was amplified as an internal reference gene to normalize the cDNA quantity added to each reaction. The gene expression levels were calculated using the 2^-ΔΔCT^ method. For each sample, three technical replicates and three biological replicates were included in the analysis. All primers used in this study are listed in Supplementary Table S2.

For the RNA-seq analysis of cotton fiber development stage, the raw data were downloaded from TM-1 genome sequencing research (Zhang et al., 2015). The clean reads were aligned to the TM-1 genome (Zhang et al., 2015) using Bowtie2 (Langmead and Salzberg, 2012). The expression of each cotton gene was derived from the read alignments and normalized to fragments per kilobase of exon model per million (FPKM) in Cufflinks (Trapnell et al., 2012).

### Sequence of *Gh_D03G1338* in the Natural Population

The full-length ORF of *Gh_D03G1338* was amplified and sequenced in 75 accessions selected from the natural population. The PCR products were ligated into a pMD18-T cloning vector (TaKaRa, Japan) for sequencing by GENEWIZ (Suzhou, China) and aligned using MEGA version 7 (Kumar et al., 2016). The cloning primers are shown in Supplementary Table S2.

## Results

### Phenotypic Variation in Fiber Length Between Natural and Segregating Populations

**FIGURE 1:**
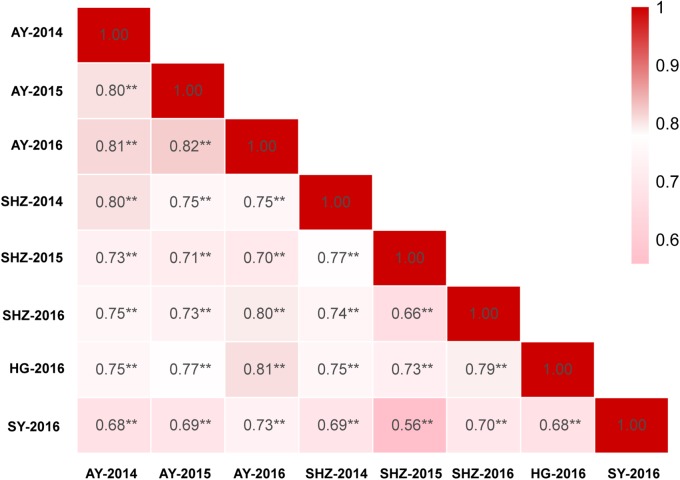
Correlation analyses of fiber length (FL) based on eight environments among 355 accessions. ^∗∗^Indicates that the correlation reached significance at 0.001.

The detailed descriptive statistics of the FL in the mapping populations are presented in Table 1. All accessions used in this study were planted in eight environments (E1: AY-2014, E2: AY-2015, E3: AY-2016, E4: SHZ-2014, E5: SHZ-2015, E6: SHZ-2016, E7: HG-2016, and E8: SY-2016) between 2014 and 2016. Extensive variation was observed in each population. In the natural population, the FL ranged from 22.60 to 35.50 mm (average 28.63 mm), whereas in the segregating population, the FL ranged from 25.90 to 33.50 mm (average 29.99 mm). The standard deviation (SD) value in the natural and segregating populations was between 0.85 and 1.74, indicating that the experimental error was small. The absolute skewness and kurtosis values of FL in all populations across all environments were <1, suggesting that the data followed an approximately normal distribution. The broad-sense heritability (*h*^2^) in the eight environments was estimated to be relatively high at 81%, suggesting that the FL was mainly controlled by genetic factors and was less affected by environmental effects. In addition, significant (*P* < 0.001) positive correlations were observed between each pair of environments, and the Pearson’s correlation coefficients ranged from 0.56 to 0.82 (Figure 1). In conclusion, the high Pearson’s correlations and stable heritability indicated that much of the FL variance was genetically controlled in the populations and suitable for a GWAS analysis and QTL mapping.

### Genome-Wide Association Study of Fiber Length Based on 355 Accessions

**FIGURE 2:**
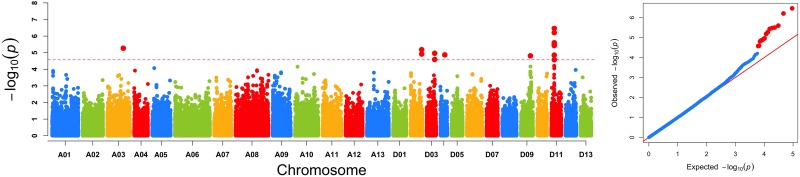
Genome-wide association scan of FL. **(A)** Manhattan plot of FL using the best linear unbiased prediction of FL value. The red dashed horizontal line represents the significance threshold (*P* < 2.5 × 10^-5^). **(B)** Quantile–quantile plots of FL.

**FIGURE 3:**
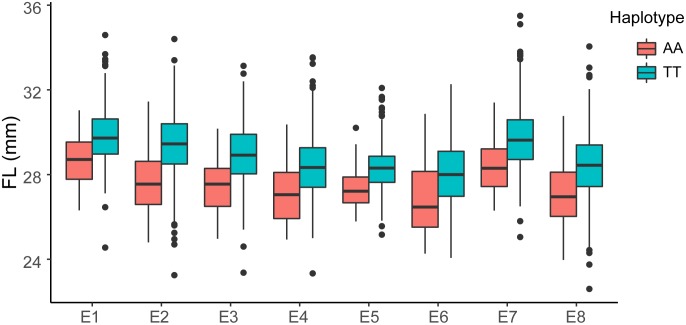
Box plots of FL based on the haplotypes of two SNPs (AA, *n* = 52 and TT, *n* = 262) on chromosome D03 in eight environments (E1: AY-2014, E2: AY-2015, E3: AY-2016, E4: SHZ-2014, E5: SHZ-2015, E6: SHZ-2016, E7: HG-2016, and E8: SY-2016). The significance of the difference was analyzed with two-tailed Student’s *t*-tests.

The GWAS population used in this study was previously described (Su et al., 2018). Briefly, 93,250 high-quality SNPs (MAF ≥ 0.05 and missing data < 0.2), including 61,618 SNPs on the At subgenome and 31,632 SNPs on the Dt subgenome, were detected among the 355 accessions on 26 chromosomes in upland cotton. The average SNP density was approximately 22.35 kb per SNP. A genome-wide association analysis of FL was performed with a mixed linear model (MLM), which greatly reduces the false-positive rates, as shown in Figure 2. Using the FL-BLUP values, 14 significant association loci were identified on six chromosomes (A03, D02, D03, D04, D09, and D11) across the eight environments, explaining 9.34–14.37% of the PV based on the *R*^2^ values (Table 2). All significant SNPs were detected in more than two environments, and 10 (71%) SNPs accounted for more than 10% of the PV (Table 2). Among the SNPs, only 1 SNP on the At subgenome and 13 SNPs on the Dt subgenome were recovered. On the At subgenome, one significant SNP (*P*-value = 5.39E-06) on chromosome A03 explaining 11.04% of the PV was detected in six environments. Notably, two significant SNPs (D03_41720764 and D03_41721072) on chromosome D03 that showed the strongest association with FL could explain 13.13–14.37% of the PV. These two SNPs generated the two haplotypes AA and TT. The accessions carrying the AA haplotype had a significantly shorter FL than those with the TT allele in the eight environments (*P* < 0.001) (Figure 3). In addition, six SNPs spanning 24.03–24.10 Mb on chromosome D11 were observed to be strongly associated with FL, explained 9.34–12.60% of the PV (Table 2). In this region, ortholog of *KRP6* (*Gh_D11G1929*), which is a member of the *KRP* family and encodes a KIP-related protein in *Arabidopsis. thaliana*. Interestingly, the expression levels of *KRP5*, which belongs to the *KRP* family, was reported to be significantly correlated with cell length in *Arabidopsis* (Wen et al., 2013). Furthermore, previous studies have demonstrated that overexpression of *Gh_D11G1929* in *Arabidopsis* produced a significantly leaf trichrome length compared with the wild type (Ma et al., 2018). From the above results, we inferred that *Gh_D11G1929* is a major gene on chromosome D11 for controlling FL and may have a potential role in the breeding process.

**Table 2:** Summary of SNPs significantly associated with fiber length.

Chromosome	Position	Major allele	Minor allele	*P*-value	*R*^2^ (%)^a^	Environment
A03	69,684,069	T	C	5.39E-06	11.04	E1, E2, E3, E6, E7, E8
D02	63,765,689	A	C	6.58E-06	10.76	E1, E3, E7, E8
D02	63,765,702	A	G	1.20E-05	10.74	E3, E4, E7, E8
D03	41,720,764	A	T	1.09E-05	13.13	E1, E2
D03	41,721,072	A	T	2.57E-05	14.37	E1, E2, E8
D04	32,246,025	T	C	1.35E-05	10.31	E1, E7
D09	41,308,487	T	C	1.55E-05	10.92	E1, E2, E3
D09	41,651,435	A	G	6.81E-05	10.37	E1, E4
D11	24,102,240	G	A	3.44E-07	12.60	E1, E2, E3, E4
D11	24,067,326	C	T	6.20E-07	9.34	E1, E2, E3, E4
D11	24,056,611	G	A	2.53E-06	9.50	E1, E2
D11	24,056,593	C	A	3.13E-06	9.54	E1, E2
D11	24,056,372	T	C	3.33E-06	11.44	E1, E2
D11	24,034,569	G	A	3.70E-06	9.70	E1, E4


*^*a*^R^*2*^ is the percentage of phenotypic variance explained by the SNP.*

### Genetic Map Construction and QTL Analysis of Fiber Length in the Segregating Generations

To further verify that these significant SNP loci associated with FL, we constructed an *F*_2_ population (198 plants in total) using two extreme accessions selected from the natural population with contrasting FLs, i.e., CRI50 (27.52 mm) and CRI60 (31.22 mm). The GBS libraries of the 198 *F*_2_ individuals and their parents were constructed for the Illumina HiSeq4000 sequencing, generating 213.31 GB of data with average depths of 33.31 and 26.94 corresponding to the two parents and the 198 offspring, respectively (Supplementary Table S3). The raw data have been deposited in the NCBI Sequence Read Archive (SRA)^2^ under accession SRP155335.

**FIGURE 4:**
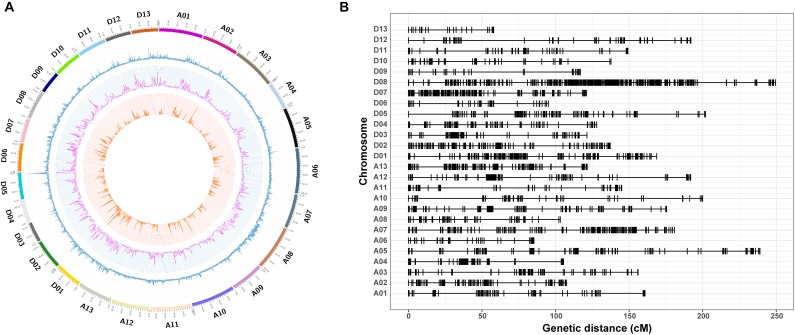
Genome-wide distribution of SNPs throughout the physical and genetic maps. **(A)** Genome-wide distribution of SNPs and genetic variants throughout the CRI50 and CRI60 genomes. The outermost box with a scale represents the 26 cotton chromosomes. The blue histogram represents the density of SNPs that are polymorphic between CRI50 and CRI60; the purple histogram indicates the density of insertions or deletions (Indels) between CRI50 and CRI60; and the orange histogram represents the density of aa × bb markers between CRI50 and CRI60. **(B)** Genetic map constructed by SNP markers.

In total, 20,698 aa × bb genotypes of SNP markers were used to screen the 198 *F*_2_ individuals, accounting for 36.76% of all 56,299 SNP markers (Figure 4A). After filtering for significant segregation distortion (*P* < 0.001) and with more than 40% missing data, 5,280 SNP markers (9.38%) were used to construct the final genetic map (Table 3 and Figure 4B). The total genetic map spanned a cumulative distance of 3,848.29 cM across 26 linkage groups with an average marker interval of 1.41 cM. The At subgenome contained 2,316 markers and covered 1,971.87 cM, whereas the Dt subgenome harbored 2,964 markers and spanned 1,876.41 cM. The SNP markers were unevenly distributed on the 26 chromosomes of upland cotton, in accordance with previous reports (Li C. et al., 2016; Zhang et al., 2016). The highest number of markers was identified on chromosome D08 (809) with an average density of 3.25 markers/cM, while the lowest number of markers (48), with an average density of 0.83 markers/cM, was found on chromosome D13. The length of the linkage groups varied from 57.96 cM on chromosome D13 to 249.28 cM on chromosome D08. The average marker interval was the lowest on chromosome A10 (0.43) and the highest on chromosome D08 (3.25 cM).

**Table 3:** Summary of the high-density genetic map.

Chromosome	SNP number	Length (cM)	Average interval (cM)	Max gap (cM)
A01	152	160.78	0.95	16.2
A02	211	107.55	1.96	27.74
A03	153	156.12	0.98	23.46
A04	164	105.45	1.56	23.36
A05	220	238.96	0.92	24.22
A06	68	85.28	0.8	12.98
A07	406	180.77	2.25	16.11
A08	101	103.21	0.98	23.8
A09	170	175.44	0.97	23.61
A10	85	199.99	0.43	14.32
A11	66	144.98	0.46	15.26
A12	226	191.84	1.18	24.93
A13	294	121.51	2.42	20.39
At	2316	1971.87	1.17	20.49
D01	444	168.78	2.63	14.31
D02	360	137.14	2.63	18.13
D03	149	121.38	1.23	15.53
D04	168	127.94	1.31	19.75
D05	188	201.95	0.93	24.14
D06	54	95.09	0.57	29.21
D07	392	121.06	3.24	13.27
D08	809	249.28	3.25	18.26
D09	50	116.93	0.44	20.87
D10	95	137.71	0.69	23.35
D11	102	149.18	0.68	6.79
D12	105	192.02	0.55	20.39
D13	48	57.96	0.83	11.78
Dt	2964	1876.41	1.67	18.14
Total	5280	3848.29	1.41	19.31


In total, 13 QTLs were identified on 10 chromosomes (A05, A06, A09, D01, D02, D03, D05, D07, D08, and D13) across the *F*_2_ and *F*_2:3_ generations (Table 4). Among the 13 QTLs, 4 QTLs were identified in the *F*_2_ generation, and 9 QTLs were identified in the *F*_2:3_ generation. Each QTL explained 1.71–22.03% of the phenotypic variation, and the LOD scores ranged from 2.51 to 10.44. Notably, 2 QTLs (qFL-D03-1/2 and qFL-D08-1/2) were detected in both the *F*_2_ and *F*_2:3_ generations and had relatively high PV, ranging from 16.52–22.03% to 5.27–7.31%, with LOD scores of 8.76–10.44 and 3.97–5.42, respectively. These results indicate that chromosomes D03 and D08 are rich in genes that potentially function in controlling cotton FL development.

**Table 4:** Stable QTLs responsible for fiber length identified in the *F*_2_ and *F*_2:3_ populations.

QTL	Generation	Chromosome	Position (cM)	LOD	*R*^2^ (%)
qFL-A05-1	*F*_2:3_	A05	170	3.18	4.42
qFL-A06-1	*F*_2:3_	A06	33	2.63	3.56
qFL-A06-2	*F*_2_	A06	47	2.51	2.59
qFL-A09-2	*F*_2:3_	A09	55	2.53	4.79
qFL-D01-1	*F*_2_	D01	58	3.01	4.42
qFL-D02-1	*F*_2_	D02	70	2.63	3.64
qFL-D03-1	*F*_2:3_	D03	95	10.44	22.03
qFL-D03-2	*F*_2_	D03	94	8.76	16.52
qFL-D05-1	*F*_2:3_	D05	52	2.59	1.71
qFL-D07-1	*F*_2:3_	D07	31	4.97	5.12
qFL-D08-1	*F*_2:3_	D08	111	5.42	7.31
qFL-D08-2	*F*_2_	D08	110	3.97	5.27
qFL-D13-1	*F*_2_	D13	20	2.61	3.44


### Identification of a Candidate Gene Potentially Underlying Fiber Length on Chromosome D03

**FIGURE 5:**
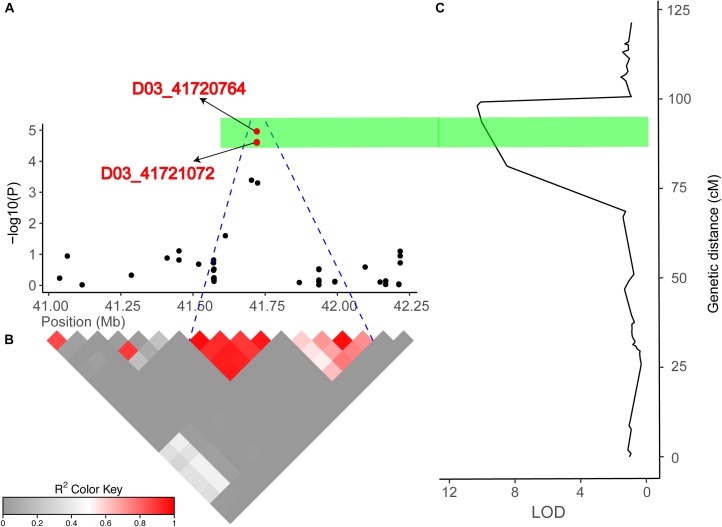
Combined results of the association mapping and linkage analysis of chromosome D03. **(A)** Peak region (41.03–42.21 Mb) on chromosome D03. **(B)** Pairwise LD between the SNP markers is indicated as *D*′ values, where dark red indicates a value of 1, and gray indicates a value of 0. The blue dotted line shown in **(A)** indicates the LD blocks that contain two significant SNPs (red dots). **(C)** The right part is the linkage map and the results of the linkage mapping, and the green bar indicates overlapping regions across the GWAS and linkage mapping results.

To screen for reliable QTLs that can be used in gene function analyses and MAS, we compared the results of the GWAS and linkage mapping. According to the physical position of the SNP markers, an overlapping region on chromosome D03 was detected in both the GWAS analysis and linkage mapping that could explain the relatively high PV of 13.75 and 19.28%, respectively, indicating that a major gene may be responsible for FL in this genomic region (Figure 5). Based on the cotton gene annotation database^3^, 26 candidate genes contained in a significant LD block region on chromosome D03 (41.52–41.91 Mb) were identified (Supplementary Table S4). Among these genes, 22 candidate genes had annotation information, while that on four candidate genes was unknown. Interestingly, *Gh_D03G1338* (*F2KP*) is involved in sucrose synthesis (Li et al., 2009), which has been reported to play a critical role in the process of fiber cell elongation. Furthermore, the expression levels of 26 putative candidate genes were analyzed using RNA-seq data obtained during the cotton (TM-1) fiber development stage downloaded from NCBI SRA under accession number PRJNA248163. In total, 11 (42.31%) genes (*Gh_D03G1316, Gh_D03G1318, Gh_D03G1319, Gh_D03G1325, Gh_D03G1326, Gh_D03G1330, Gh_D03G1331, Gh_D03G1332, Gh_D03G1337, Gh_D03G1338*, and *Gh_D03G1339*) exhibited higher expression levels from fiber-5 dpa to fiber-20 dpa than other genes (Supplementary Figure S1). To confirm the accuracy of the RNA-seq analysis, we selected *Gh_D03G1316, Gh_D03G1318, Gh_D03G1325, Gh_D03G1330, Gh_D03G1332*, and *Gh_D03G1339* and performed a qRT-PCR analysis to test the reliability of the transcription levels. The qRT-PCR results of these six genes showed trends similar to those observed in the deep sequencing data (Supplementary Figure S2).

### Haplotype Analysis of *Gh_D03G1338* in the Natural Population

**FIGURE 6:**
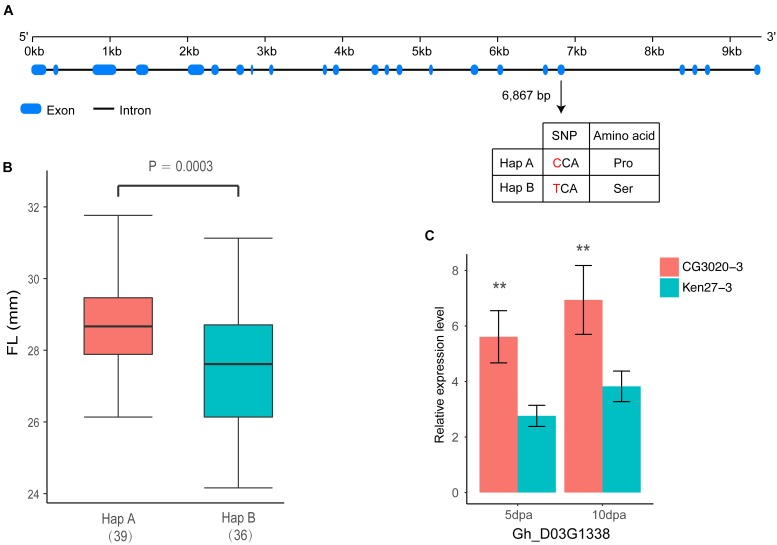
Identification of the FL causal gene *Gh_D03G1338* on chromosome D03. **(A)** Exon–intron structure of *Gh_D03G1338* and the polymorphism in two haplotypes with the ‘C’ and ‘T’ alleles. **(B)** Box plot of FL based on the two haplotypes mentioned above (*n* = 39 versus 36). Differences between the haplotypes were analyzed by two-tailed Student’s *t*-tests. **(C)** Comparison of expression levels of *Gh_D03G1338* during fiber development stages by qRT-PCR (^∗∗^indicates significance at the 0.001 probability level).

Among these 26 genes, *Gh_D03G1338* stood out as an *F2KP* homolog in the cotton genome. *F2KP* is a key regulator of carbohydrate metabolism in all eukaryotes (Draborg et al., 2001). In total, 21 exons have been found in *Gh_D03G1338*, which is similar to *F2KP* identified in *Arabidopsis* (Figure 6A). In addition, one non-synonymous variation between two haplotypes leads to a single amino acid substitution of proline (Pro) to serine (Ser) on exon 19 located in the histidine phosphatase superfamily domain by cloning the full coding region of *Gh_D03G1338* from 75 accessions selected from natural populations (Figure 6A). The varieties carrying haplotype A had positive phenotypic effects on the FL and showed a longer FL than the varieties carrying haplotype B (*P* = 0.0003) (Figure 6B). Further confirming the effect of this gene on FL, according to the qRT-PCR analysis, *Gh_D03G1338* had a significantly higher expression in fiber development stages (5 and 10 dpa) of CG3020-3 (long fiber varieties) than that of Ken27-3 (short fiber varieties) (Figure 6C).

## Discussion

Single-nucleotide polymorphisms are the most abundant genetic variants in crop genomes (Rafalski, 2002; Ganal et al., 2009; McNally et al., 2009). The rapid development of next-generation sequencing technologies has led to tremendous progress in the development of numerous SNP markers for GWAS analyses and QTL mapping in various crop species, including rice (Han et al., 2016), maize (Li X. et al., 2016), cotton (Su et al., 2018), and soybean (Sonah et al., 2015). To date, the application of SNP genotyping technology has successfully accelerated genetic research, revealing loci controlling various traits in cotton, including fiber quality (Li C. et al., 2016), plant architecture (Su et al., 2018), disease resistance (Li T. et al., 2017), and other important agronomic traits (Jia et al., 2016). In addition, analyses of SNP haplotypes based on natural populations can be utilized to improve the selection of favorable alleles in breeding programs (Wen et al., 2015). In this study, over 90,000 high-quality SNPs were identified in a diverse set of 355 cotton accessions, and a high-density genetic map was constructed by GBS-seq using 5,280 SNP markers. Subsequently, GWAS and linkage analyses were successfully applied to FL with a greatly improved marker density (22.35 Kb/SNP for the GWAS and 0.73 cM/SNP for the biparents) compared with that of traditional SSR markers (Jamshed et al., 2016; Nie et al., 2016). Most importantly, the low cost of using SNPs could effectively reduce labor-intensive and time-consuming processes, providing a convenient and effective alternative for the identification of molecular markers for MAS breeding in the future.

Heritability is a main factor that significantly affects the accuracy of QTL analyses (Bernardo, 2004). As expected, most mapped QTLs corresponded to these characteristics with better genetic determination or stable heritability (Said et al., 2015). Generally, a heritability value < 20% is considered low, and a value > 50% is considered high (Stanfield, 1983). In our research, the broad-sense heritability (*h*^2^) of FL was estimated to be relatively high at 81%. Similar results have been found in previous studies, and Wang et al. (2015), Jamshed et al. (2016) and Jia et al. (2016) also showed broad-sense heritabilities of 70, 93, and 89%, respectively. Although FL has a relatively high broad-sense heritability, due to the environmental instability of traits, detecting more reproducible QTLs in a few environments is challenging. Therefore, we performed a genome-wide association analysis in eight environments (four locations over 3 years) to detect stable QTLs under multiple environmental conditions and improve the accuracy of the QTLs in the genetic analysis of FL. All 14 significant loci detected in the present study were detected in more than two single environments and the combined analysis (FL-BLUP). Four loci, namely, D02_63765689, D02_63765702, D11_24102240, and D11_24067326, were detected in four environments and the combined analysis. Among these loci, D11_24102240 and D11_24067326 were also identified in the same location (Anyang) over three continuous years (2015–2016), suggesting that D11_24102240 and D11_24067326 have environmental specificity and great potential for improving FL in the cotton-growing area of the Yellow River region. Notably, one stable QTL on chromosome A03 was detected in five environments, providing an opportunity to apply this locus to MAS breeding to improve fiber quality in various planting areas. In the present study, the normal distribution and high heritability of FL variations were detected in both the natural and biparental populations, suggesting that FL in cotton is a highly polygenic trait and is suitable for QTL analyses.

Previous reports have demonstrated that coexisting At/Dt subgenomes have been under asymmetric domestication and the Dt subgenomes have a substantial influence in determining fiber quality (Fang et al., 2014; Wang et al., 2017). Over 1,000 fiber quality QTLs have been summarized in the CottonQTLdb database, and 455 (58%) QTLs have been identified in the Dt subgenome (Said et al., 2015). Previous comparative meta-analyses conducted by Rong et al. (2007), Lacape et al. (2010) and Said et al. (2013) also indicated that more QTLs responsible for fiber quality-related traits reside in the Dt subgenome, which has a greater impact than the At subgenome. Furthermore, more fiber quality-related genes, including *GhPIS* (Long et al., 2018), *GhMML4* (Wu et al., 2018), and *GhFL2* (Ma et al., 2018) on chromosomes D01, D08, and D11, respectively, have been identified in the Dt subgenome. Similarly, among the 27 QTLs identified in this study, only a few QTLs (19%) were identified in the At subgenome, and 22 (81%) QTLs were identified in the Dt subgenome. This result is consistent with that of previous reports and further supports the hypothesis that the Dt subgenome plays a more important role in determining fiber quality.

In the present study, we detected 14 and 13 QTLs for FL in the natural and biparental populations, respectively. To further screen for QTLs that can be used in MAS and gene cloning with a high accuracy, high stability, and smaller confidence intervals, we compared our results with published research according to the SSR markers (Shen et al., 2007; Zhang et al., 2012; Shao et al., 2014). Three reliable QTLs located on chromosome D02 and D03 were reported in previous studies. Two SNPs (D02_63765689 and D02_63765702) on chromosome D02 were mapped to an adjacent region of BNL2485, which was named qFL03.1 by Shao et al. (2014). qFL-D02-1 identified in 70 cM on chromosome D02 overlapped between NAU990 and NAU1529, which was reported by Shen et al. (2007). In particular, a tightly linked region including D03_41720764 and D03_41721072 was mapped in the vicinity of the common QTL qFL-D03-1/qFL-D03-2 in the *F*_2_ and *F*_2:3_ generations and could explain more than 15% of the observed PV. Furthermore, this region was also near NAU2297 as described by Zhang et al. (2012). Thus, these stable QTLs that are responsible for FL may provide valuable information for cotton breeders using MAS, and these findings provide validation that combining the results of GWAS with traditional QTL mapping can increase confidence in the identity of the main QTLs in cotton research.

Over the past decade, numerous studies have reported that several important pathways, such as plant hormone (auxin, gibberellin, and brassinosteroid) signaling (Xiao et al., 2010; Chen et al., 2012), Ca^2+^/K^+^ transporters (Ruan et al., 2001; Huang et al., 2008), vacuolar invertase (Wang and Ruan, 2010) and sucrose synthase (Li et al., 2009), play critical roles in the process of rapid fiber cell elongation and have a significant effect on the molecular mechanisms associated with FL. The locus on chromosome D03 detected in both the GWAS analysis and linkage mapping could explain more than 10% of the variation in cotton FL, and 26 genes were located in a tightly linked LD block interval from 41.52 to 41.91 Mb (Figure 5). Moreover, GWAS analysis showed that haplotype TT had a significant increase in FL across multiple environments in this region (Figure 3). Therefore, we wonder whether there is a major gene associated with FL control that plays a determinate role in this genomic region. After carefully analyzing the 26 genes in this region, we found that the functional annotations of four of these genes are unknown, and three of them are highly expressed during fiber development stage compared with other genes (Supplementary Figure S1). However, the qRT-PCR analysis of these four genes did not significantly differ between the long fiber and short fiber varieties (Supplementary Figure S3). *Gh_D03G1338* caught our attention based on the gene annotation of cotton, namely, this gene encodes the bifunctional enzyme fructose-6-phosphate 2-kinase (*F2KP*) with two functional kinase domains. The ortholog of *F2KP* in *A. thaliana* is a key factor affecting photosynthetic carbon partitioning between sucrose and starch during photosynthesis (Draborg et al., 2001), and sucrose is the initial substrate required for cellulose synthesis and plays an important role in the development of cotton fibers (Haigler et al., 2001, 2007; Xu et al., 2012). The results of the blast alignment show that the coding sequence identity of *Gh_D03G1338* with the gene of *F2KP* is as high as 46%, and *Gh_D03G1338* encodes a protein sharing 77.60% sequence identity with the *F2KP* protein. A non-synonymous SNP that caused a change from C (39 cultivars) to T (36 cultivars) at 6,867 bp in the genome region resulted in a change from proline to serine at amino acid position 1,011 in the histidine phosphatase superfamily domain (Figure 6A). Furthermore, HapA was significantly associated with increased FL (*P* = 0.0003) (Figure 6B). The RNA-seq data showed that this gene was highly expressed during fiber development from 5 to 20 days post anthesis (dpa) (Supplementary Figure S1). Then, we selected two haplotype varieties (CC and TT) for a qRT-PCR analysis. At fiber-5 dpa and fiber-10 dpa, *Gh_D03G1338* in the long fiber varieties with CC was more highly expressed than that in the short fiber varieties with TT (Figure 6C). Hence, we speculate that this gene may promote cotton fiber elongation by regulating the synthesis of sucrose and diverting more carbon to fiber growth. These results imply that *Gh_D03G1338* is the most likely candidate gene responsible for the QTL that controls the FL trait on chromosome D03.

## Author Contributions

SY, JH, and LL designed the experiments. CZ, QL, and LG collected the accessions. CZ and LL performed the experiments and wrote the manuscript. HlW and HtW revised the language. All authors read and approved the final manuscript.

## Conflict of Interest Statement

The authors declare that the research was conducted in the absence of any commercial or financial relationships that could be construed as a potential conflict of interest.

## Footnotes

1 https://github.com/YinLiLin/R-CMplot

2 https://www.ncbi.nlm.nih.gov/sra/

3 http://www.cottongen.org
