# Supplementary material for: Identification of Loci and Candidate Genes Responsible for Fiber Length in Upland Cotton (Gossypium hirsutum L.) via Association Mapping and Linkage Analyses
Source: Front Plant Sci. 2019 Feb 5;10:53. doi: 10.3389/fpls.2019.00053 (PMC6370998; doi:10.3389/fpls.2019.00053)
Supplement: Supplementary file 1 [file Data_Sheet_1.PDF]

Supplementary Material

Identification of loci and candidate genes responsible for fiber length in upland cotton (*Gossypium hirsutum* L.) via association mapping and linkage analyses

Chi Zhang#, Libei Li#, Qibao Liu

#Contributed equally to this work.

*** Correspondence:** Shuxun Yu**:** ysx195311@163.com.

Supplementary Tables

Supplementary Table 1: Summary of the 355 SLAF-seq accessions.

Sample	Cultivar/Germplasm line	Origin	Ecological region	Clean reads	Read length	Clean Bases (Mb)	Mapping rate (%)	Mean depth
S1	Zhong2191	Henan	YRR	2,060,776	80	329.7	98.94	4.57
S2	Zhong40418	Henan	YRR	1,953,983	80	312.6	99.31	4.33
S3	Zhong40618	Henan	YRR	2,602,031	80	416.3	99.25	5.77

S4	Zhong40612	Henan	YRR	2,753,449	80	440.6	99.14	6.11
S5	Zhong51811	Henan	YRR	2,120,626	80	339.3	99.18	4.70
S6	CRI94A1822	Henan	YRR	2,119,866	80	339.2	99.31	4.70
S7	Zhong61832	Henan	YRR	2,447,539	80	391.6	98.54	5.43
S8	Zhong71239	Henan	YRR	2,306,633	80	369.1	98.70	5.12
S9	PB12-1-10	Henan	YRR	2,455,469	80	392.9	99.15	5.45
S10	PB12-1-7	Henan	YRR	3,109,997	80	497.6	98.87	6.90
S11	PB12-1-8	Henan	YRR	2,240,263	80	358.4	98.89	4.97
S12	Zhong1476	Henan	YRR	2,023,692	80	323.8	99.13	4.49
S13	Zhong151222	Henan	YRR	2,060,750	80	329.7	99.07	4.57
S14	Zhong152201	Henan	YRR	2,419,621	80	387.1	99.00	5.37
S15	Zhong152201SQ	Henan	YRR	2,432,241	80	389.2	99.18	5.40
S16	Zhong152214	Henan	YRR	2,954,591	80	472.7	99.05	6.55
S17	Zhong152224	Henan	YRR	2,312,093	80	369.9	99.17	5.13
S18	SS2011	Henan	YRR	2,656,266	80	425	99.14	5.89
S19	CQ2012-3	Henan	YRR	2,648,525	80	423.8	99.34	5.88
S20	CQ2012-4	Henan	YRR	2,613,289	80	418.1	99.37	5.80
S21	Zhong29T41	Henan	YRR	2,159,647	80	345.5	99.13	4.79
S22	Zhong29T42	Henan	YRR	2,601,951	80	416.3	98.96	5.77

S23	L426	Henan	YRR	1,719,364	80	275.1	99.08	3.81
S24	K640	Shandong	YRR	2,127,164	80	340.3	99.13	4.72
S25	N82	America	Amerasian	1,554,657	80	248.7	98.89	3.45
S26	P21-6-7	America	Amerasian	2,618,266	80	418.9	99.30	5.81
S27	Shannong SF06	Shandong	YRR	2,003,835	80	320.6	99.00	4.45
S28	sGK16	Beijing	YRR	2,445,710	80	391.3	99.28	5.43
S29	V321-20-14	Beijing	YRR	2,282,204	80	365.2	99.35	5.06
S30	Baimian 17	Henan	YRR	2,228,620	80	356.6	99.45	4.94
S31	Chaoaoyang 1	Azerbaijan	Amerasian	2,147,802	80	343.6	99.49	4.76
S32	Deltapine 20	America	Amerasian	1,835,450	80	293.7	98.85	4.07
S33	De 97-047	America	Amerasian	2,269,305	80	363.1	99.07	5.03
S34	Guannong 1	Liaoning	NSER	2,032,094	80	325.1	99.47	4.51
S35	Han2490	Hebei	YRR	2,153,636	80	344.6	99.45	4.78
S36	Han656	Hebei	YRR	2,691,400	80	430.6	99.13	5.97
S37	Han559	Hebei	YRR	2,341,165	80	374.6	99.42	5.19
S38	Han667	Hebei	YRR	2,416,789	80	386.7	99.22	5.36
S39	Han686	Hebei	YRR	2,541,363	80	406.6	98.93	5.64
S40	Han9609	Hebei	YRR	2,274,857	80	364	99.28	5.05
S41	Heishanmian1	Liaoning	NSER	2,761,341	80	441.8	98.92	6.13

S42	Jinmian3	Liaoning	NSER	1,786,724	80	285.9	98.85	3.96
S43	Jinmian10	Shanxi	YRR	2,313,590	80	370.2	99.28	5.13
S44	Jinmian21	Shanxi	YRR	2,300,224	80	368	99.34	5.10
S45	Jinmian23	Shanxi	YRR	2,838,263	80	454.1	99.08	6.30
S46	Jinmian5	Shanxi	YRR	2,224,189	80	355.9	98.93	4.93
S47	Liaomian10	Liaoning	NSER	1,960,546	80	313.7	98.87	4.35
S48	Liaomian17	Liaoning	NSER	1,921,278	80	307.4	98.82	4.26
S49	Liaomian5	Liaoning	NSER	2,488,613	80	398.2	98.76	5.52
S50	Liaomian6	Liaoning	NSER	1,884,525	80	301.5	98.27	4.18
S51	Liaomian7	Liaoning	NSER	2,017,239	80	322.8	98.32	4.48
S52	Liaomian9	Liaoning	NSER	2,041,945	80	326.7	98.86	4.53
S53	Liaoyangduanjie	Liaoning	NSER	2,092,310	80	334.8	98.83	4.64
S54	Lu154	Shandong	YRR	1,962,866	80	314.1	98.53	4.36
S55	Lumianyan19	Shandong	YRR	2,182,657	80	349.2	98.81	4.84
S56	Lumian2153	Shandong	YRR	2,278,007	80	364.5	98.91	5.05
S57	Nongken5	the North of Xinjiang	NIR	2,411,688	80	385.9	98.90	5.35
S58	Shan70	Shanxi	NIR	3,159,325	80	505.5	98.41	7.01
S59	Shizao1	Hebei	YRR	1,973,542	80	315.8	98.59	4.38

S60	Shizao2	Hebei	YRR	2,604,364	80	416.7	99.00	5.78
S61	Shizao3	Hebei	YRR	2,729,196	80	436.7	97.14	6.06
S62	X13-7	Henan	YRR	1,801,755	80	288.3	99.09	4.00
S63	X25TF	Henan	YRR	2,785,260	80	445.6	98.90	6.18
S64	Xiazao1	Hebei	YRR	2,347,939	80	375.7	99.32	5.21
S65	Xiazao2	Hebei	YRR	2,631,398	80	421	99.02	5.84
S66	Xiazao3	Hebei	YRR	2,192,213	80	350.8	99.07	4.86
S67	Xinluzao11	the North of Xinjiang	NIR	2,276,120	80	364.2	98.24	5.05
S68	Xinluzao36	the North of Xinjiang	NIR	2,050,178	80	328	98.58	4.55
S69	Xinluzao3	the North of Xinjiang	NIR	3,075,952	80	492.2	98.50	6.82
S70	Xinluzao42	the North of Xinjiang	NIR	2,496,194	80	399.4	99.11	5.54
S71	Xinluzao45	the North of Xinjiang	NIR	2,353,151	80	376.5	99.26	5.22
S72	Xinluzao4	the North of Xinjiang	NIR	2,550,152	80	408	98.84	5.66

S73	Xinluzao6	the North of Xinjiang	NIR	2,243,926	80	359	99.11	4.98
S74	Xinluzao8	the North of Xinjiang	NIR	2,081,070	80	333	98.73	4.62
S75	Xinluzao9	the North of Xinjiang	NIR	1,550,469	80	248.1	98.51	3.44
S76	Mianxiang368	Henan	YRR	1,903,697	80	304.6	98.84	4.22
S77	Yu1335	Henan	YRR	2,254,186	80	360.7	98.69	5.00
S78	Yumian12	Henan	YRR	3,339,607	80	534.3	99.09	7.41
S79	Yuzao8E13	Henan	YRR	2,187,787	80	350	99.20	4.85
S80	Yuzao9110	Henan	YRR	2,187,114	80	349.9	98.84	4.85
S81	Jinmian57	Shanxi	YRR	2,010,206	80	321.6	98.55	4.46
S82	YunzaoN95	Shanxi	YRR	1,991,584	80	318.7	98.94	4.42
S83	Zhong416	Henan	YRR	2,071,272	80	331.4	98.89	4.60
S84	Zhong425-5	Henan	YRR	2,697,952	80	431.7	99.09	5.99
S85	Zhong716	Henan	YRR	2,479,472	80	396.7	98.74	5.50
S86	Zhong213	Henan	YRR	2,169,109	80	347.1	98.22	4.81
S87	Zhongchuang 88	Hebei	YRR	2,055,165	80	328.8	98.93	4.56
S88	CRI10	Henan	YRR	2,330,037	80	372.8	99.12	5.17

S89	CRI14	Henan	YRR	2,186,627	80	349.9	99.16	4.85
S90	CRI16	Henan	YRR	2,402,710	80	384.4	98.43	5.33
S91	CRI20	Henan	YRR	2,596,649	80	415.5	98.50	5.76
S92	CRI24	Henan	YRR	2,753,249	80	440.5	99.03	6.11
S93	CRI27	Henan	YRR	2,188,073	80	350.1	98.98	4.85
S94	CRI30	Henan	YRR	2,394,526	80	383.1	98.69	5.31
S95	CRI36	Henan	YRR	2,042,230	80	326.8	98.96	4.53
S96	Han256	Hebei	YRR	2,239,447	80	358.3	99.19	4.97
S97	CRI37	Henan	YRR	2,192,488	80	350.8	99.07	4.86
S98	CRI42	Henan	YRR	2,974,180	80	475.9	98.76	6.60
S99	CRI50	Henan	YRR	2,120,164	80	339.2	98.79	4.70
S100	CRI58	Henan	YRR	2,615,766	80	418.5	98.90	5.80
S101	CRI64	Henan	YRR	2,016,377	80	322.6	98.88	4.47
S102	CRI74	Henan	YRR	2,098,420	80	335.7	98.75	4.65
S103	Zhong00776	Henan	YRR	1,967,754	80	314.8	99.01	4.37
S104	Zhong20398	Henan	YRR	2,335,762	80	373.7	98.86	5.18
S105	Zhong61930	Henan	YRR	2,211,613	80	353.9	98.94	4.91
S106	Zhong61995	Henan	YRR	3,185,194	80	509.6	99.23	7.07
S107	Zhong102909	Henan	YRR	2,485,723	80	397.7	98.81	5.51

S108	Zhong103026	Henan	YRR	2,371,442	80	379.4	98.84	5.26
S109	Zhong103028	Henan	YRR	2,706,886	80	433.1	98.59	6.01
S110	Zhong103030	Henan	YRR	2,677,363	80	428.4	98.90	5.94
S111	Zhong103032	Henan	YRR	2,155,295	80	344.8	98.57	4.78
S112	Zhong103075	Henan	YRR	3,009,454	80	481.5	98.89	6.68
S113	Zhong103164	Henan	YRR	2,274,353	80	363.9	99.11	5.05
S114	Zhong103425	Henan	YRR	2,506,229	80	401	98.98	5.56
S115	Zhong103297	Henan	YRR	1,836,023	80	293.8	99.22	4.07
S116	Zhong109056	Henan	YRR	1,665,370	80	266.5	98.89	3.70
S117	CPB12-1-7	Henan	YRR	2,312,046	80	369.9	98.53	5.13
S118	CPB12-1-9	Henan	YRR	2,482,277	80	397.2	98.96	5.51
S119	CPB12-2-7	Henan	YRR	3,014,242	80	482.3	98.73	6.69
S120	298	the North of Xinjiang	NIR	2,387,342	80	382	98.88	5.30
S121	Zhong602186	Henan	YRR	1,864,278	80	298.3	99.11	4.14
S122	CG3020-1	America	Amerasian	3,186,780	80	509.9	99.25	7.07
S123	CG3020-3	America	Amerasian	2,435,262	80	389.6	99.09	5.40
S124	FM1735	America	Amerasian	1,885,782	80	301.7	99.31	4.18
S125	G2005	Henan	YRR	1,491,430	80	238.6	99.05	3.31

S126	Zhongzhimian2	Beijing	YRR	2,870,246	80	459.2	99.20	6.37
S127	H109	Henan	YRR	2,660,223	80	425.6	98.97	5.90
S128	H559	Henan	YRR	2,089,191	80	334.3	99.35	4.64
S129	LIH33	Henan	YRR	1,900,420	80	304.1	99.04	4.22
S130	Phy-7	America	Amerasian	1,799,886	80	288	98.99	3.99
S131	STS458	America	Amerasian	1,592,488	80	254.8	98.90	3.53
S132	TM-1	America	Amerasian	2,108,321	80	337.3	99.12	4.68
S133	Aoshimian 6	Beijing	YRR	2,220,893	80	355.3	98.15	4.93
S134	Baimian985	Henan	YRR	2,104,291	80	336.7	98.32	4.67
S135	Cang198	Hebei	YRR	3,052,698	80	488.4	99.37	6.77
S136	CBB	Henan	YRR	2,189,323	80	350.3	98.98	4.86
S137	CNB	Henan	YRR	1,962,345	80	314	98.34	4.35
S138	Deltapine14	America	Amerasian	2,295,588	80	367.3	99.45	5.09
S139	Deltapine15	America	Amerasian	2,248,562	80	359.8	98.36	4.99
S140	Fanmian3	Henan	YRR	2,701,339	80	432.2	98.97	5.99
S141	Ganzao109	Jiangxi	YZRR	3,847,035	80	615.5	99.21	8.53
S142	Guoxinmian11	Hebei	YRR	1,823,838	80	291.8	98.20	4.05
S143	Han7860	Hebei	YRR	2,289,879	80	366.4	99.21	5.08
S144	Ji4025	Hebei	YRR	2,250,374	80	360.1	98.99	4.99

S145	Jimian 26	Shanxi	YRR	2,299,667	80	367.9	99.40	5.10
S146	BM03	Henan	YRR	1,945,972	80	311.4	99.11	4.32
S147	Kelin098	Henan	YRR	1,994,201	80	319.1	98.72	4.42
S148	Liaomiao23	Liaoning	NSER	2,356,994	80	377.1	99.18	5.23
S149	Liaomiao27	Liaoning	NSER	2,804,089	80	448.7	99.03	6.22
S150	Liaomiao28	Liaoning	NSER	2,352,265	80	376.4	99.01	5.22
S151	Lu05R59	Shandong	YRR	2,285,561	80	365.7	99.08	5.07
S152	Lu7619	Shandong	YRR	1,901,060	80	304.2	99.06	4.22
S153	Luyanmian17	Shandong	YRR	2,032,447	80	325.2	98.98	4.51
S154	Luyanmian21	Shandong	YRR	2,370,562	80	379.3	99.26	5.26
S155	Luyanmian28	Shandong	YRR	2,609,036	80	417.4	99.15	5.79
S156	Luyanmian36	Shandong	YRR	2,841,174	80	454.6	98.78	6.30
S157	Luyanmian38	Shandong	YRR	2,574,112	80	411.9	99.14	5.71
S158	Miaobao21	Shandong	YRR	2,802,634	80	448.4	99.15	6.22
S159	Renhe39	Shandong	YRR	2,040,901	80	326.5	98.55	4.53
S160	Rihuimian6	Anhui	YZRR	2,146,860	80	343.5	99.02	4.76
S161	ShannongSF01	Shandong	YRR	2,365,525	80	378.5	99.18	5.25
S162	Shan79	Shanxi	NIR	2,390,243	80	382.4	99.23	5.30
S163	Stoneville 2B	America	Amerasian	3,095,123	80	495.2	99.30	6.87

S164	Xinmian 33B	America	Amerasian	1,978,348	80	316.5	98.73	4.39
S165	Xinzhimian5	Henan	YRR	2,260,016	80	361.6	99.04	5.01
S166	Yinhuashu	Shandong	YRR	1,671,667	80	267.5	98.34	3.71
S167	You009	Henan	YRR	1,694,312	80	271.1	98.36	3.76
S168	Zhong109	Henan	YRR	2,024,945	80	324	98.67	4.49
S169	CRI17	Henan	YRR	1,870,723	80	299.3	98.59	4.15
S170	CRI19	Henan	YRR	2,235,614	80	357.7	98.87	4.96
S171	CRI43	Henan	YRR	2,339,569	80	374.3	98.70	5.19
S172	CRI60	Henan	YRR	2,071,966	80	331.5	99.16	4.60
S173	Zhong662	Henan	YRR	2,608,463	80	417.4	99.01	5.79
S174	Zhong679	the North of Xinjiang	NIR	2,440,338	80	390.5	98.85	5.41
S175	Zhong69	Henan	YRR	2,404,367	80	384.7	98.99	5.33
S176	Zhong800319	Shandong	YRR	2,135,457	80	341.7	98.61	4.74
S177	CRI 94A915	Henan	YRR	3,236,814	80	517.9	99.11	7.18
S178	CRI12	Henan	YRR	2,184,636	80	349.5	98.88	4.85
S179	CRI35	Henan	YRR	1,698,335	80	271.7	99.16	3.77
S180	CRI41	Henan	YRR	2,046,761	80	327.5	98.56	4.54
S181	CRI45	Henan	YRR	2,500,633	80	400.1	98.86	5.55

S182	CRI49	Henan	YRR	2,658,685	80	425.4	98.63	5.90
S183	CRI7	Henan	YRR	2,239,455	80	358.3	98.92	4.97
S184	Zhongzhimian 8	Beijing	YRR	2,481,233	80	397	98.88	5.50
S185	Zhongzhimian GD89	Beijing	YRR	2,879,680	80	460.7	99.29	6.39
S186	Shan920346	Shanxi	NIR	2,191,772	80	350.7	99.13	4.86
S187	XianⅢ9704	America	Amerasian	2,067,262	80	330.8	98.97	4.59
S188	US-1	America	Amerasian	2,597,533	80	415.6	99.08	5.76
S189	Kyrgyzstan cotton	Kyrgyzstan	Amerasian	1,723,817	80	275.8	99.08	3.82
S190	Bazhou5409	the North of Xinjiang	NIR	1,617,575	80	258.8	99.12	3.59
S191	Yumian1	Sichuan	YZRR	1,489,456	80	238.3	98.88	3.30
S192	Huazhong910102	Hubei	YZRR	2,686,230	80	429.8	99.45	5.96
S193	Israel cotton	Israel	Amerasian	1,934,199	80	309.5	98.82	4.29
S194	Ken6614	the North of Xinjiang	NIR	2,177,249	80	348.4	99.09	4.83
S195	Ken0074	the North of Xinjiang	NIR	1,877,754	80	300.4	99.10	4.17
S196	Chuan239-1	Sichuan	YZRR	2,462,800	80	394	99.12	5.46
S197	Bamian3	Sichuan	YZRR	2,400,963	80	384.2	99.11	5.33

S198	Chuan338	Sichuan	YZRR	3,409,881	80	545.6	99.23	7.57
S199	Chuan267	Sichuan	YZRR	2,419,796	80	387.2	99.04	5.37
S200	Chuan65	Sichuan	YZRR	2,286,267	80	365.8	98.49	5.07
S201	Chuanjian1	Sichuan	YZRR	2,431,550	80	389	99.33	5.39
S202	Xinluzao 2	the North of Xinjiang	NIR	2,358,522	80	377.4	99.38	5.23
S203	Xinluzao10	the North of Xinjiang	NIR	2,238,295	80	358.1	99.23	4.97
S204	Xinluzao12	the North of Xinjiang	NIR	2,746,908	80	439.5	98.87	6.09
S205	Xinluzao13	the North of Xinjiang	NIR	3,030,604	80	484.9	99.13	6.72
S206	Xinluzao15	the North of Xinjiang	NIR	2,618,205	80	418.9	99.21	5.81
S207	Xinluzao16	the North of Xinjiang	NIR	2,888,084	80	462.1	99.15	6.41
S208	Xinluzao17	the North of Xinjiang	NIR	1,969,069	80	315.1	98.84	4.37
S209	Xinluzao18	the North of	NIR	3,762,407	80	602	99.23	8.35

		Xinjiang						
S210	Xinluzao19	the North of Xinjiang	NIR	2,177,805	80	348.4	99.05	4.83
S211	Xinluzao20	the North of Xinjiang	NIR	2,802,952	80	448.5	99.42	6.22
S212	Xinluzao21	the North of Xinjiang	NIR	2,051,858	80	328.3	99.21	4.55
S213	Xinluzao22	the North of Xinjiang	NIR	2,173,493	80	347.8	98.79	4.82
S214	Xinluzao23	the North of Xinjiang	NIR	3,089,548	80	494.3	98.98	6.85
S215	Xinluzao24	the North of Xinjiang	NIR	2,535,770	80	405.7	99.33	5.63
S216	Yumian5	Henan	YRR	3,314,343	80	530.3	99.16	7.35
S217	Yumian18	Henan	YRR	3,364,121	80	538.3	99.18	7.46
S218	Yumian21	Henan	YRR	2,284,159	80	365.5	99.11	5.07
S219	Yun1729	Shanxi	YRR	2,054,954	80	328.8	99.33	4.56
S220	Zhemian11	Zhejiang	YZRR	1,856,073	80	297	99.29	4.12
S221	Tkuo	the North of	NIR	2,017,749	80	322.8	99.18	4.48

		Xinjiang						
S222	Bo425	the North of Xinjiang	NIR	2,932,437	80	469.2	99.08	6.51
S223	Xinluzhong 60	the South of Xinjiang	NIR	3,670,948	80	587.4	99.44	8.14
S224	Ken27-3	the North of Xinjiang	NIR	1,979,731	80	316.8	98.70	4.39
S225	B-3	the South of Xinjiang	NIR	2,633,165	80	421.3	99.29	5.84
S226	Xinluzao25	the North of Xinjiang	NIR	3,383,108	80	541.3	99.31	7.51
S227	Xinluzao26	the South of Xinjiang	NIR	2,214,142	80	354.3	99.18	4.91
S228	Xinluzao27	the North of Xinjiang	NIR	1,528,067	80	244.5	98.71	3.39
S229	Xinluzao28	the North of Xinjiang	NIR	2,637,433	80	422	98.93	5.85
S230	Xinluzao29	the North of Xinjiang	NIR	1,991,211	80	318.6	99.06	4.42

S231	Xinluzao30	the North of Xinjiang	NIR	1,791,761	80	286.7	98.84	3.98
S232	Xinluzao32	the North of Xinjiang	NIR	2,254,336	80	360.7	98.73	5.00
S233	Xinluzao33	the North of Xinjiang	NIR	3,041,271	80	486.6	98.90	6.75
S234	Xinluzao34	the North of Xinjiang	NIR	1,951,565	80	312.3	98.94	4.33
S235	Xinluzao35	the North of Xinjiang	NIR	2,734,383	80	437.5	99.10	6.07
S236	Xinluzao37	the North of Xinjiang	NIR	2,640,335	80	422.5	98.97	5.86
S237	Xinluzao38	the North of Xinjiang	NIR	3,745,405	80	599.3	98.93	8.31
S238	Xinluzao39	the North of Xinjiang	NIR	2,375,896	80	380.1	99.02	5.27
S239	Xinluzao40	the North of Xinjiang	NIR	2,908,309	80	465.3	99.09	6.45
S240	Xinluzao41	the South of	NIR	4,427,036	80	708.3	99.12	9.82

		Xinjiang						
S241	Xinluzao46	the North of Xinjiang	NIR	3,514,635	80	562.3	99.20	7.80
S242	Xinluzao47	the North of Xinjiang	NIR	2,189,811	80	350.4	99.25	4.86
S243	Xinluzao48	the North of Xinjiang	NIR	1,921,437	80	307.4	98.93	4.26
S244	Xinluzao49	the North of Xinjiang	NIR	3,132,896	80	501.3	99.31	6.95
S245	Xinluzao50	the North of Xinjiang	NIR	3,322,562	80	531.6	99.20	7.37
S246	Xinluzao51	the North of Xinjiang	NIR	2,944,507	80	471.1	99.22	6.53
S247	Xinluzhong1	the South of Xinjiang	NIR	2,331,282	80	373	98.87	5.17
S248	Xinluzhong3	the South of Xinjiang	NIR	2,688,261	80	430.1	98.29	5.96
S249	Xinluzhong4	the North of Xinjiang	NIR	2,658,473	80	425.4	99.03	5.90

S250	Xinluzhong5	the North of Xinjiang	NIR	3,186,587	80	509.9	99.24	7.07
S251	Xinluzhong6	the South of Xinjiang	NIR	1,822,421	80	291.6	99.14	4.04
S252	Xinluzhong7	the South of Xinjiang	NIR	2,521,645	80	403.5	99.35	5.59
S253	Xinluzhong8	the South of Xinjiang	NIR	4,232,214	80	677.2	99.38	9.39
S254	Xinluzhong9	the South of Xinjiang	NIR	3,320,197	80	531.2	99.20	7.37
S255	Xinluzhong10	the South of Xinjiang	NIR	2,411,200	80	385.8	98.85	5.35
S256	Xinluzhong12	the South of Xinjiang	NIR	3,451,116	80	552.2	99.25	7.66
S257	Xinluzhong13	the South of Xinjiang	NIR	2,176,758	80	348.3	99.21	4.83
S258	Xinluzhong14	the South of Xinjiang	NIR	2,059,834	80	329.6	98.85	4.57
S259	Xinluzhong15	the South of	NIR	2,393,228	80	382.9	99.11	5.31

		Xinjiang						
S260	Xinluzhong16	the South of Xinjiang	NIR	2,932,213	80	469.2	99.20	6.51
S261	Xinluzhong17	the South of Xinjiang	NIR	2,246,577	80	359.5	99.10	4.98
S262	Xinluzhong19	the South of Xinjiang	NIR	1,977,391	80	316.4	99.10	4.39
S263	Xinluzhong20	the North of Xinjiang	NIR	2,131,218	80	341	98.89	4.73
S264	Xinluzhong21	the South of Xinjiang	NIR	3,028,797	80	484.6	99.16	6.72
S265	Xinluzhong22	the South of Xinjiang	NIR	3,354,892	80	536.8	99.24	7.44
S266	Xinluzhong26	the South of Xinjiang	NIR	3,099,715	80	496	99.11	6.88
S267	Xinluzhong27	the South of Xinjiang	NIR	2,504,491	80	400.7	99.22	5.56
S268	Xinluzhong28	the South of Xinjiang	NIR	2,410,563	80	385.7	99.18	5.35

S269	Xinluzhong30	the South of Xinjiang	NIR	3,584,085	80	573.5	99.28	7.95
S270	Xinluzhong32	the South of Xinjiang	NIR	1,899,631	80	303.9	99.17	4.21
S271	Xinluzhong34	the South of Xinjiang	NIR	3,134,370	80	501.5	99.21	6.95
S272	Xinluzhong35	the South of Xinjiang	NIR	1,751,161	80	280.2	99.17	3.89
S273	Xinluzhong40	the South of Xinjiang	NIR	3,524,903	80	564	98.91	7.82
S274	Xinluzhong41	the South of Xinjiang	NIR	2,400,664	80	384.1	99.25	5.33
S275	Xinluzhong45	the South of Xinjiang	NIR	2,428,727	80	388.6	99.35	5.39
S276	Xinluzhong46	the South of Xinjiang	NIR	3,785,837	80	605.7	99.49	8.40
S277	Xinluzhong47	the South of Xinjiang	NIR	2,372,994	80	379.7	99.15	5.26
S278	Kangcaoganlin	America	Amerasian	2,864,029	80	458.2	99.17	6.35

S279	Jiangyin1	Yangtze River	YZRR	2,109,763	80	337.6	99.07	4.68
S280	Huihe36	the South of Xinjiang	NIR	1,359,385	80	217.5	98.75	3.02
S281	Jinken1042	the North of Xinjiang	NIR	2,171,821	80	347.5	98.90	4.82
S282	Guoxinmian9	Hebei	YRR	2,687,027	80	429.9	99.42	5.96
S283	Junmian1	the South of Xinjiang	NIR	2,579,292	80	412.7	99.29	5.72
S284	Huiyuan717	the North of Xinjiang	NIR	3,459,289	80	553.5	99.43	7.67
S285	Yunzao219	Shanxi	YRR	2,763,682	80	442.2	99.25	6.13
S286	Yunzao33-356	Shanxi	YRR	1,792,808	80	286.8	99.31	3.98
S287	Jinmian2	Liaoning	NSER	2,832,725	80	453.2	99.11	6.28
S288	Chaoyangmian2	Liaoning	NSER	1,942,406	80	310.8	99.23	4.31
S289	Dunhuang77-116	Gansu	NIR	2,992,599	80	478.8	98.99	6.64
S290	Ganmian4	Gansu	NIR	3,848,095	80	615.7	99.26	8.54
S291	Guannongzao C-50	Liaoning	NSER	3,032,659	80	485.2	98.99	6.73
S292	Guannongchangzao B14	Liaoning	NSER	2,551,615	80	408.3	99.06	5.66
S293	Yanzao1	Jiangsu	YZRR	3,568,903	80	571	99.24	7.92

S294	Yanzao2	Jiangsu	YZRR	3,246,007	80	519.4	99.37	7.20
S295	Jinmian6	Liaoning	NSER	2,963,188	80	474.1	98.81	6.57
S296	Jinken69-2	the North of Xinjiang	NIR	2,467,762	80	394.8	99.23	5.47
S297	Jinken148-39	the North of Xinjiang	NIR	2,906,196	80	465	99.13	6.45
S298	Zhuangjiahan102	the North of Xinjiang	NIR	2,175,629	80	348.1	99.26	4.83
S299	Yinshan4	Henan	YRR	3,338,133	80	534.1	98.94	7.41
S300	Ejing1	Hebei	YRR	3,383,330	80	541.3	99.06	7.51
S301	Simian3	Sichuan	YZRR	2,766,398	80	442.6	99.21	6.14
S302	Stoneville 4B	America	Amerasian	1,749,809	80	280	98.83	3.88
S303	Jimian10	Hebei	YRR	2,026,299	80	324.2	99.00	4.50
S304	Jimian11	Hebei	YRR	2,052,221	80	328.4	99.25	4.55
S305	Jimian12	Hebei	YRR	2,052,712	80	328.4	99.21	4.55
S306	Jimian16	Hubei	YZRR	2,106,206	80	337	99.33	4.67
S307	Jimian17	Hubei	YZRR	2,883,841	80	461.4	99.35	6.40
S308	Sumian4	Jiangsu	YZRR	1,982,137	80	317.1	98.97	4.40
S309	Ekangmian2	Hebei	YRR	3,794,508	80	607.1	99.21	8.42

S310	Ekangmian3	Hubei	YZRR	4,098,607	80	655.8	99.01	9.09
S311	Ekangmian6	Hubei	YZRR	2,175,132	80	348	99.09	4.83
S312	Edaimian	Hebei	YRR	1,614,903	80	258.4	98.88	3.58
S313	Xuzhou142	Jiangsu	YZRR	2,402,323	80	384.4	99.02	5.33
S314	Sumian9	Jiangsu	YZRR	2,571,488	80	411.4	98.96	5.70
S315	Sumian12	Jiangsu	YZRR	1,907,942	80	305.3	99.20	4.23
S316	Sukang191	Jiangsu	YZRR	2,194,639	80	351.1	99.10	4.87
S317	Gangmian1	Hebei	YRR	1,967,668	80	314.8	99.22	4.37
S318	Gangmian2	Hebei	YRR	2,801,620	80	448.3	99.27	6.22
S319	Coker 201	America	Amerasian	3,019,394	80	483.1	99.13	6.70
S320	Daihongdai	Hunan	YZRR	1,968,229	80	314.9	98.76	4.37
S321	Yishuhong	Hubei	YZRR	2,679,883	80	428.8	99.18	5.95
S322	Bomian1	Shandong	YRR	2,521,232	80	403.4	99.29	5.59
S323	Kemian4	Jiangsu	YZRR	3,019,528	80	483.1	99.20	6.70
S324	Ganmian11	Jiangxi	YZRR	3,187,679	80	510	99.27	7.07
S325	Ganmian12	Jiangxi	YZRR	3,288,790	80	526.2	99.39	7.30
S326	Xianmian13	Hunan	YZRR	2,172,337	80	347.6	99.20	4.82
S327	Yapengmian	Hubei	YZRR	2,000,487	80	320.1	99.30	4.44
S328	Jijiaodezimian	Jiangsu	YZRR	2,308,072	80	369.3	99.27	5.12

S329	611bo	Tajikistan	Amerasian	2,427,735	80	388.4	99.21	5.39
S330	Annong121	Anhui	YZRR	1,996,145	80	319.4	99.12	4.43
S331	Deltapine16	America	Amerasian	1,548,695	80	247.8	99.19	3.44
S332	Delfos 531	America	Amerasian	2,320,456	80	371.3	99.23	5.15
S333	Dunmian1	Gansu	NIR	3,368,716	80	539	99.23	7.47
S334	Dunmian2	Gansu	NIR	2,984,132	80	477.5	99.28	6.62
S335	Ganmian2	Jiangxi	YZRR	2,656,531	80	425	99.47	5.89
S336	Ganmina3	Jiangxi	YZRR	3,087,716	80	494	99.32	6.85
S337	Ganmian47	Jiangxi	YZRR	2,202,092	80	352.3	99.49	4.89
S338	Guangyedaizimina	America	Amerasian	2,121,541	80	339.4	99.44	4.71
S339	Ji668	Hebei	YRR	2,883,048	80	461.3	99.43	6.40
S340	Jimian25	Hebei	YRR	2,128,188	80	340.5	99.14	4.72
S341	Jinmian5	Liaoning	NSER	2,982,497	80	477.2	99.28	6.62
S342	Keke1543	Uzbekistan	Amerasian	1,904,328	80	304.7	99.10	4.22
S343	Ningmian1	Jiangsu	YZRR	3,199,904	80	512	99.22	7.10
S344	Ningmian22	Jiangsu	YZRR	3,327,208	80	532.4	99.23	7.38
S345	Nongda94-7	Hebei	YRR	2,443,298	80	390.9	99.25	5.42
S346	Nongdamian8	Hebei	YRR	3,551,974	80	568.3	99.31	7.88
S347	Shumian1	Sichuan	YZRR	4,252,235	80	680.4	98.75	9.43

S348	Sumian1	Jiangsu	YZRR	1,951,194	80	312.2	99.28	4.33
S349	Sumian22	Jiangsu	YZRR	2,417,921	80	386.9	99.25	5.36
S350	Xiangmian10	Hunan	YZRR	2,595,860	80	415.3	99.22	5.76
S351	Xuzhou219	Jiangsu	YZRR	3,034,410	80	485.5	99.28	6.73
S352	Yinshan8	Henan	YRR	2,740,436	80	438.5	99.27	6.08
S353	Yumian1	Henan	YRR	2,839,258	80	454.3	99.09	6.30
S354	Yumian2	Henan	YRR	4,474,962	80	716	99.35	9.93
S355	Xinluzao53	the North of Xinjiang	NIR	2,923,626	80	467.8	99.05	6.49

Supplementary Table 2: List of all primers used in this study.

Primer	Sequence (5'~ 3')
QGh_D03G1316-1	TTTCTCGCTTGGTTTTCCCG
QGh_D03G1316-2	GGCTGAGGTGAAACGAGGTC
QGh_D03G1318-1	TGCTTTACTCTTCGGGGACG
QGh_D03G1318-2	GCGTACACCATGACAGCCAA
QGh_D03G1319-1	GCATGATCTTAATGCTTCTGTTGTG
QGh_D03G1319-2	CCGAGTTCCCAACATTTCCG

QGh_D03G1325-1	TAAAGCCAAAACCGAATCCACAA
QGh_D03G1325-2	CTGAATTGCCTCTGATTTGGCG
QGh_D03G1326-1	GGAGGAATGGAGGCGAACAA
QGh_D03G1326-2	ATATGCCGACGAGAGCGAAG
QGh_D03G1328-1	TTGTGAAATGTTCGAAATAAAGCTG
QGh_D03G1328-2	TCAACACTTGCACCCTTGC
QGh_D03G1330-1	GAGAGCGTGCGCAGGTAATG
QGh_D03G1330-2	CCACATTTTTCCGTGCTCTTGT
QGh_D03G1332-1	TATAGTGGCGCACAAATGCC
QGh_D03G1332-2	AACCTGTCCAAGTCCCCTTT
QGh_D03G1337-1	ACAACAACCCCATTGCTTGC
QGh_D03G1337-2	CTTTTCTCTCCGGCCACTGT
QGh_D03G1338-1	TTGTCGAGGAGGGTCCAAAC
QGh_D03G1338-2	GCGATACCCTGTCCGCTTTA
QGh_D03G1339-1	CTCGTCATCCTCTTCGTCCTG
QGh_D03G1339-2	AGGAAGATCGGCTCCTTTGG
Actin-F	ATCCTCCGTCTTGACCTTG
Actin-R	TGTCCGTCAGGCAACTCAT
D03G1338-1	CACGGGGGACTCTAGAATGGGGACGGGAACATCGAG

D03G1338-2	GATCGGGGAAATTCGAGCTCTCAATCCATTAGTTTGTATCTTTTC

Supplementary Table 3: Sequencing statistics of CRI50, CRI60 and 198 F2 families.

sample	clean reads	mapped reads	mapping rate(%)	Sequence depth coverage	Genome base coverage(%)
Z50R_1	48,342,150	48,215,113	99.74	32.05	9.12
Z60R_1	50,936,242	50,814,375	99.76	34.57	9.01
07_12	6,319,996	6,312,306	99.88	12.78	2.60
07_14	8,787,230	8,776,014	99.87	14.16	3.41
07_17	8,107,224	8,099,139	99.90	13.56	3.35
07_19	7,216,494	7,209,278	99.90	13.59	2.94
07_20	8,234,824	8,226,406	99.90	13.69	3.26
07_22	6,801,644	6,793,714	99.88	12.29	3.02
07_26	6,849,232	6,839,084	99.85	12.31	2.94
07_27	7,570,282	7,560,881	99.88	13.61	3.15
07_31	6,747,348	6,734,637	99.81	11.81	2.95
07_35	8,322,650	8,312,705	99.88	13.12	3.54
07_36	7,921,566	7,911,813	99.88	13.74	3.24

07_38	8,738,552	8,729,461	99.90	13.94	3.41
07_39	5,952,952	5,946,306	99.89	11.92	2.73
07_41	6,533,484	6,524,705	99.87	12.01	3.01
07_43	6,788,068	6,774,594	99.80	12.68	3.03
07_45	7,093,526	7,084,262	99.87	12.63	3.11
07_46	6,589,362	6,580,010	99.86	12.00	3.03
07_47	6,084,806	6,078,043	99.89	12.09	2.79
07_8	8,380,086	8,371,060	99.89	14.17	3.40
10_10	6,825,000	6,797,630	99.60	11.58	3.17
10_14	7,563,864	7,531,775	99.58	11.56	3.36
10_15	7,417,502	7,383,035	99.54	11.56	3.31
10_22	7,911,068	7,869,588	99.48	12.75	3.29
10_23	7,763,266	7,671,887	98.82	12.09	3.29
10_31	7,435,392	7,402,128	99.55	12.45	3.12
10_32	7,553,458	7,493,960	99.21	12.29	3.22
10_33	7,891,318	7,858,046	99.58	13.01	3.27
10_34	7,075,898	7,049,385	99.63	12.09	3.05
10_35	6,407,486	6,383,103	99.62	11.37	3.01
10_36	7,220,572	7,189,573	99.57	10.67	3.60

10_37	2,858,294	2,855,269	99.89	6.86	1.79
10_38	7,934,692	7,892,230	99.46	13.34	3.27
10_39	7,840,472	7,800,726	99.49	13.19	3.31
10_4	6,024,406	5,986,876	99.38	11.40	2.84
10_40	7,341,772	7,309,309	99.56	12.29	3.21
10_41	7,826,518	7,793,820	99.58	13.64	3.18
10_42	7,053,206	7,023,174	99.57	12.81	3.01
10_43	6,281,480	6,253,223	99.55	11.88	2.78
10_44	7,267,012	7,234,795	99.56	14.10	2.94
10_45	6,925,066	6,899,773	99.63	12.47	3.04
10_46	5,499,546	5,480,220	99.65	11.27	2.56
10_5	6,806,880	6,781,912	99.63	13.20	2.85
10_9	2,596,066	2,593,029	99.88	6.30	1.60
11_11	6,517,292	6,507,332	99.85	11.11	3.01
11_12	6,611,430	6,604,105	99.89	11.78	3.07
11_14	6,605,414	6,587,458	99.73	11.60	3.01
11_17	6,851,794	6,838,270	99.80	12.90	3.03
11_18	7,257,164	7,247,593	99.87	12.93	3.11
11_20	7,070,088	7,061,948	99.88	12.99	3.11

11_21	7,100,582	7,090,825	99.86	12.80	3.13
11_22	8,151,998	8,142,143	99.88	13.01	3.37
11_23	6,976,334	6,968,392	99.89	12.63	2.98
11_24	6,383,992	6,375,872	99.87	11.84	3.04
11_25	6,480,604	6,473,506	99.89	11.04	3.07
11_26	7,224,250	7,209,111	99.79	12.43	3.18
11_27	7,621,906	7,611,412	99.86	12.63	3.30
11_28	7,088,818	7,080,700	99.89	12.46	3.07
11_29	6,156,254	6,146,210	99.84	12.81	2.84
11_3	6,932,192	6,900,055	99.54	12.05	3.07
11_30	7,540,314	7,529,417	99.86	12.82	3.20
11_31	6,957,060	6,944,108	99.81	12.09	3.09
11_32	6,620,826	6,612,297	99.87	11.97	2.98
11_33	6,807,018	6,798,773	99.88	12.59	3.09
11_34	8,486,312	8,476,463	99.88	13.46	3.62
11_36	6,557,034	6,550,114	99.89	12.45	3.00
11_37	7,666,008	7,656,119	99.87	12.37	3.47
11_39	6,383,786	6,376,527	99.89	12.17	2.99
11_4	7,155,982	7,128,361	99.61	11.59	3.29

11_40	6,392,150	6,385,009	99.89	11.41	3.04
11_5	7,345,622	7,296,069	99.33	12.72	3.05
11_7	6,220,524	6,212,867	99.88	11.41	2.93
11_8	6,561,840	6,551,554	99.84	11.91	3.05
11_9	6,748,968	6,741,858	99.89	13.01	2.91
12_10	6,105,770	6,099,510	99.90	11.82	2.82
12_11	7,240,036	7,230,026	99.86	14.07	3.01
12_15	6,601,122	6,593,601	99.89	12.45	3.05
12_16	5,606,580	5,595,194	99.80	11.48	2.71
12_17	7,295,570	7,287,149	99.88	13.11	3.14
12_20	6,058,892	6,037,268	99.64	12.82	2.77
12_21	6,534,290	6,523,586	99.84	12.05	2.74
12_23	6,171,106	6,161,523	99.84	11.15	2.83
12_24	5,641,784	5,635,360	99.89	10.75	2.75
12_26	5,893,076	5,886,146	99.88	12.19	2.62
12_27	6,381,168	6,372,987	99.87	11.16	2.84
12_28	6,191,734	6,174,853	99.73	11.57	2.84
12_30	5,879,472	5,868,633	99.82	11.03	2.70
12_31	6,140,240	6,132,435	99.87	11.65	2.86

12_32	6,283,972	6,273,702	99.84	11.15	2.85
12_33	6,880,592	6,867,539	99.81	12.33	3.06
12_34	5,762,510	5,753,946	99.85	10.91	2.80
12_36	7,057,716	7,051,634	99.91	13.02	2.89
12_37	5,264,966	5,259,880	99.90	10.82	2.45
12_38	4,745,946	4,740,583	99.89	9.86	2.46
12_39	5,298,688	5,292,879	99.89	10.33	2.56
12_40	6,308,096	6,301,507	99.90	11.16	2.91
12_7	7,204,822	7,195,266	99.87	12.94	3.16
12_9	6,339,394	6,330,296	99.86	12.32	2.96
15_10	6,685,328	6,673,163	99.82	11.73	3.06
15_12	6,739,884	6,734,315	99.92	11.99	3.07
15_13	5,218,404	5,214,063	99.92	10.16	2.62
15_15	5,947,952	5,940,410	99.87	11.01	2.86
15_16	6,019,646	6,012,685	99.88	11.65	2.82
15_17	6,137,474	6,130,820	99.89	11.15	2.91
15_21	6,255,714	6,247,614	99.87	11.81	2.86
15_22	6,070,158	6,063,787	99.90	11.62	2.80
15_27	5,097,938	5,092,063	99.88	10.10	2.50

15_29	4,769,366	4,765,171	99.91	10.29	2.41
15_3	5,908,418	5,901,052	99.88	10.52	2.83
15_30	6,400,228	6,393,896	99.90	12.05	2.89
15_31	5,552,642	5,547,695	99.91	11.20	2.59
15_33	6,394,862	6,387,885	99.89	11.81	2.88
15_38	6,737,668	6,731,520	99.91	11.56	3.12
15_39	7,210,024	7,204,486	99.92	13.54	2.82
15_4	4,443,218	4,438,825	99.90	9.33	2.27
15_40	7,086,622	7,079,355	99.90	12.40	3.05
15_42	7,161,880	7,156,762	99.93	13.20	2.95
15_44	8,127,210	8,120,585	99.92	14.63	3.07
15_45	6,375,488	6,359,340	99.75	11.65	2.77
15_6	5,625,168	5,613,329	99.79	10.65	2.65
15_7	6,707,210	6,700,377	99.90	11.53	3.05
15_8	6,446,688	6,439,460	99.89	11.43	2.95
15_9	5,796,292	5,784,271	99.79	11.03	2.68
17_10	9,010,778	9,002,137	99.90	15.38	3.32
17_11	8,943,758	8,936,256	99.92	15.98	3.13
17_12	7,184,346	7,177,937	99.91	13.60	2.80

17_13	6,571,292	6,565,662	99.91	12.40	2.87
17_15	6,523,496	6,517,041	99.90	12.16	2.79
17_16	7,483,044	7,476,626	99.91	13.06	3.05
17_18	6,989,380	6,981,635	99.89	12.65	2.93
17_19	8,205,788	8,197,655	99.90	14.09	3.09
17_21	8,488,588	8,480,885	99.91	14.47	3.18
17_22	8,630,796	8,622,578	99.90	14.46	3.23
17_23	9,120,558	9,110,825	99.89	15.38	3.26
17_25	6,227,352	6,222,736	99.93	11.92	2.71
17_26	8,195,188	8,187,896	99.91	15.13	3.07
17_27	8,665,262	8,655,082	99.88	15.43	3.22
17_28	6,220,018	6,214,851	99.92	12.73	2.68
17_29	8,142,514	8,135,332	99.91	15.02	3.09
17_30	8,676,072	8,666,978	99.90	16.23	3.12
17_35	8,191,430	8,184,061	99.91	14.42	3.16
17_36	6,323,750	6,316,290	99.88	11.90	2.82
17_37	6,595,984	6,586,718	99.86	13.49	2.81
17_38	6,721,262	6,714,085	99.89	12.85	2.96
17_39	5,401,296	5,393,391	99.85	11.15	2.56

17_4	7,241,486	7,235,594	99.92	12.66	3.05
17_40	7,019,608	7,011,391	99.88	12.63	3.06
17_47	8,454,242	8,443,745	99.88	13.33	3.54
17_5	8,312,630	8,297,745	99.82	13.64	3.19
17_50	7,089,876	7,081,733	99.89	12.72	3.12
17_7	8,744,272	8,736,458	99.91	15.39	3.20
17_8	8,727,598	8,712,662	99.83	14.85	3.21
17_9	8,883,866	8,873,767	99.89	15.52	3.26
8_13	7,869,786	7,836,845	99.58	13.66	3.23
8_14	9,542,734	9,512,462	99.68	13.31	3.79
8_21	8,190,180	8,161,519	99.65	12.81	3.50
8_24	6,946,436	6,915,273	99.55	11.54	3.21
8_3	7,806,654	7,759,844	99.40	13.71	3.07
8_30	8,023,172	7,993,892	99.64	11.91	3.78
8_31	8,687,750	8,664,571	99.73	12.57	3.72
8_34	8,449,446	8,422,722	99.68	12.42	3.79
8_35	8,410,684	8,348,512	99.26	12.71	3.66
8_36	8,937,690	8,908,286	99.67	14.22	3.53
8_4	9,219,142	9,184,467	99.62	12.55	4.02

8_46	7,182,940	7,156,460	99.63	12.00	3.11
8_6	8,113,694	8,094,255	99.76	11.44	3.82
9_10	7,640,254	7,624,237	99.79	11.69	3.41
9_11	7,232,864	7,216,376	99.77	11.73	3.22
9_13	8,034,964	8,013,008	99.73	12.76	3.41
9_14	7,844,526	7,828,231	99.79	12.26	3.43
9_15	6,432,856	6,422,383	99.84	10.70	3.09
9_16	7,063,950	7,051,801	99.83	11.74	3.30
9_17	8,126,102	8,110,123	99.80	12.43	3.66
9_18	6,367,190	6,356,529	99.83	10.60	3.13
9_19	7,626,328	7,608,817	99.77	12.37	3.44
9_20	7,742,678	7,725,928	99.78	12.44	3.49
9_21	6,610,242	6,597,548	99.81	11.45	3.17
9_25	7,028,532	7,015,242	99.81	11.64	3.34
9_26	6,081,240	6,058,884	99.63	10.64	3.03
9_27	6,146,164	6,135,446	99.83	11.05	2.94
9_28	7,041,768	7,030,156	99.84	12.76	3.14
9_29	6,305,796	6,295,767	99.84	11.36	3.04
9_30	3,521,636	3,517,517	99.88	7.47	2.19

9_31	13,842,342	13,817,232	99.82	16.78	4.69
9_32	7,972,194	7,957,438	99.81	12.36	3.57
9_33	3,802,610	3,797,947	99.88	6.66	2.46
9_34	6,321,938	6,292,160	99.53	11.25	2.91
9_35	5,657,146	5,630,905	99.54	10.14	2.79
9_36	6,057,914	6,028,760	99.52	11.97	2.74
9_37	5,918,868	5,874,504	99.25	10.80	2.73
9_38	6,425,746	6,395,167	99.52	11.96	2.92
9_4	6,723,080	6,708,292	99.78	11.37	3.23
9_40	5,712,060	5,687,481	99.57	10.85	2.69
9_41	7,140,682	7,105,960	99.51	12.84	3.08
9_42	6,406,356	6,373,953	99.49	11.61	2.88
9_43	6,590,952	6,560,726	99.54	12.24	2.97
9_5	7,418,000	7,404,778	99.82	11.83	3.31
9_7	8,023,468	8,007,665	99.80	12.18	3.53
9_8	7,849,436	7,831,074	99.77	12.12	3.46

Supplementary Table 4: Summary of the annotations of 26 genes.

G.hirsutum gene ID	Arabidopsis ID	ArabDesc	Swissprot ID	Trembl ID	KEGG ID
Gh_D03G1316	AT5G60800	Heavy metal transport/detoxification superfamily protein		B9HQM6	
Gh_D03G1317	AT4G05160	AMP-dependent synthetase and ligase family protein	Q9M0X9	B9SCH9	ath:AT4G05160
Gh_D03G1318	AT4G05160	AMP-dependent synthetase and ligase family protein	Q9M0X9	B9SCH9	ath:AT4G05160
Gh_D03G1319	AT3G14830	unknow		I1M2X5	
Gh_D03G1320	AT1G08110	lactoylglutathione lyase family protein / glyoxalase I family protein	O04885	A0A061	pop:POPTR_749645
Gh_D03G1321	AT1G08110	lactoylglutathione lyase family protein / glyoxalase I family protein	O04885	B9SCI1	pop:POPTR_749645
Gh_D03G1322	AT1G08110	lactoylglutathione lyase family protein / glyoxalase I family protein	O04885	B9SCI1	pop:POPTR_749645
Gh_D03G1323	AT1G08110	lactoylglutathione lyase family protein /	O49818	I1LR74	pop:POPTR_749645

		glyoxalase I family protein			
Gh_D03G1324	AT3G45010	serine carboxypeptidase-like 48	Q56WF8	Q2Z1Y2	pop:POPTR_1117480
Gh_D03G1325	AT5G22950	SNF7 family protein	Q9FFB3	F6GW93	vvi:100260253
Gh_D03G1326	AT2G31490	unknow		M5Y579	
Gh_D03G1327	AT3G44990	xyloglucan endo-transglycosylase-related 8	Q9SJL9	C6TJL7	ath:AT2G36870
Gh_D03G1328	AT2G28130	unknow		D7SNB6	
Gh_D03G1329	AT3G44940	Protein of unknown function (DUF1635)		I1LR87	
Gh_D03G1330	AT1G08130	DNA ligase 1	Q42572	M5W3M2	ath:AT1G08130
Gh_D03G1331	AT2G28150	Domain of unknown function (DUF966)		B9SCJ8	
Gh_D03G1332	AT5G22920	CHY-type/CTCHY-type/RING-type Zinc finger protein	Q9CR50	B9HQP1	ath:AT5G22920
Gh_D03G1333	AT2G16030	S-adenosyl-L-methionine-dependent methyltransferases superfamily protein		B9RVC0	
Gh_D03G1334	AT2G30020	Protein phosphatase 2C family protein	O80871	B9S5C5	spu:576623
Gh_D03G1335	AT1G07150	mitogen-activated protein kinase kinase kinase 13	Q40541	M5WRK0	nve:NEMVE_v1g104385
Gh_D03G1336	AT2G30500	Kinase interacting (KIP1-like) family protein		B9SD87	mdo:100011544
Gh_D03G1337	AT1G07120	unknow	Q9LI74	B9HND5	

Gh_D03G1338	AT1G07110	fructose-2,6-bisphosphatase	Q9MB58	D7SJU7	vvi:100244966
Gh_D03G1339	AT3G16720	TOXICOS EN LEVADURA 2	Q8L9T5	D7SJU6	mdo:100028589
Gh_D03G1340	AT1G22500	RING/U-box superfamily protein	Q9SRM0	B9SQP8	dan:Dana_GF16575
Gh_D03G1341	AT2G30080	ZIP metal ion transporter family	O64738	B9HND0	

Supplementary Figures

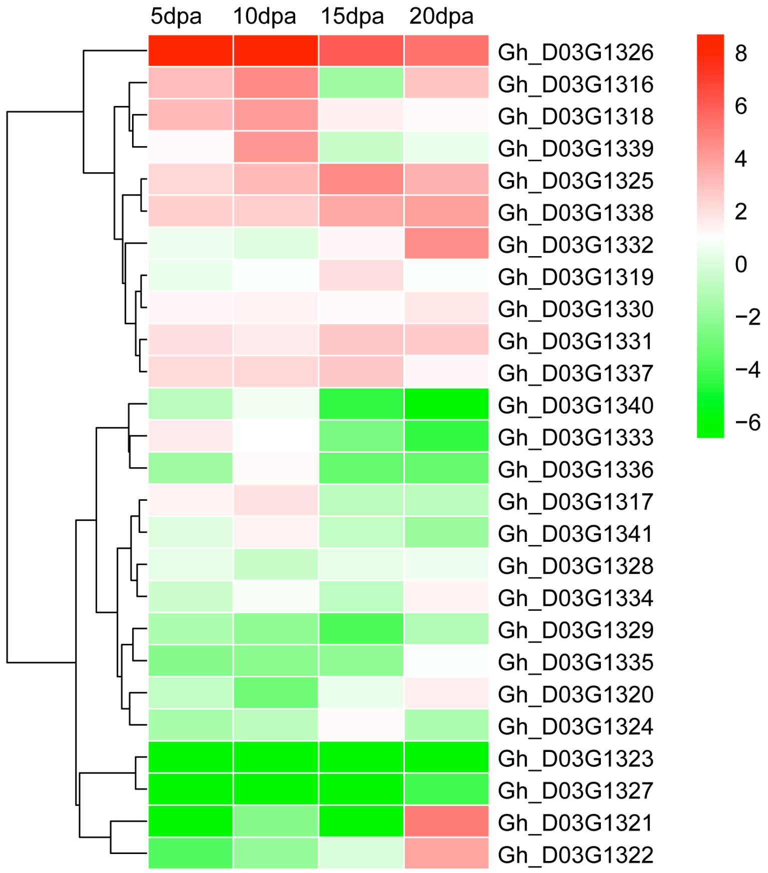

Supplementary Figure 1: Expression pattern of 26 genes related to fiber length during the fiber development stage. Red indicates high expression, and green indicates low expression.

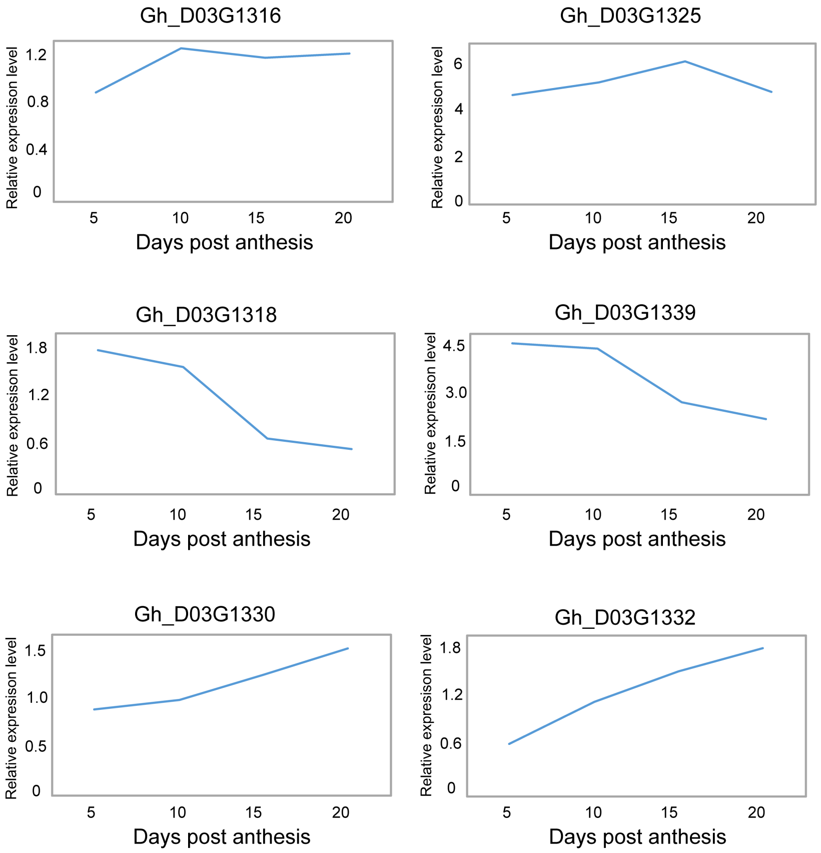

Supplementary Figure 2: Expression of genes during the fiber development stage determined by qRT-PCR.

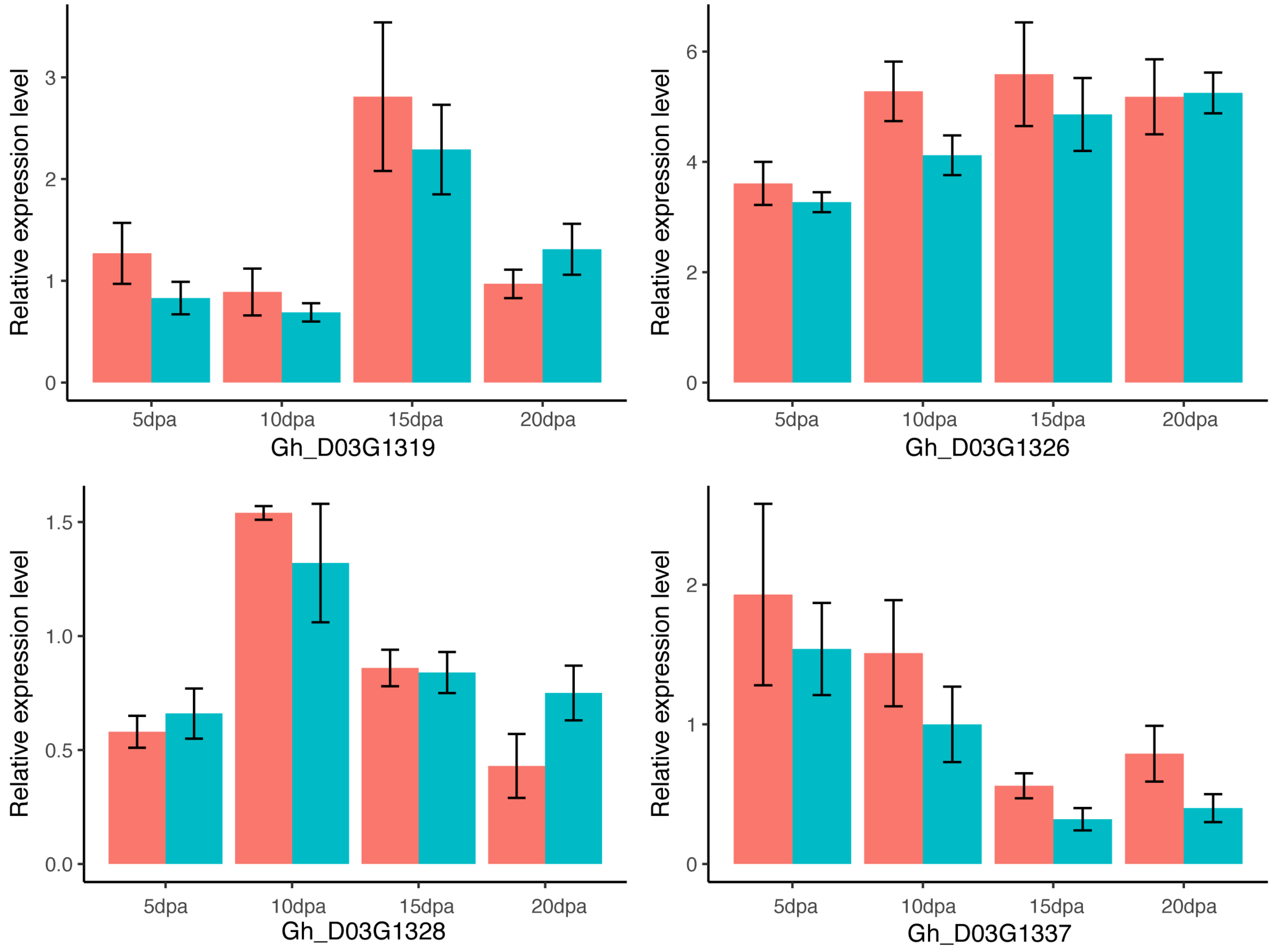

Relative expression level
0
1
2
3
5dpa
10dpa
15dpa
20dpa
Gh_D03G1319
Relative expression level
0
2
4
6
5dpa
10dpa
15dpa
20dpa
Gh_D03G1326
Relative expression level
0.0
0.5
1.0
1.5
5dpa
10dpa
15dpa
20dpa
Gh_D03G1328
Relative expression level
0
1
2
5dpa
10dpa
15dpa
20dpa
Gh_D03G1337

Supplementary Figure 3. Expression profiles of *Gh_D03G1319*, *Gh_D03G1326*, *Gh_D03G1328* and *Gh_D03G1337* between long fiber variety ‘CG3020-3’ (red) and short fiber variety ‘Ken27-3’ (green). The x-axis represents developmental stages (5, 10, 15, and 20 dpa), and the y-axis indicates the relative expression levels as determined by qRT-PCR. The error bars indicate the standard deviation of three biological replicates.
